# Microglia integrated neural spheroids enable neuroinflammatory responses and correct network dysfunction induced by alpha-synuclein mutation

**DOI:** 10.1038/s42003-026-10696-w

**Published:** 2026-07-15

**Authors:** Jiajing Zhang, Yi Wei Lim, Angelica Medina, Yu-Chi Chen, Yining Irene Wang, Martin Carrasco Carvajal, Marc Ferrer, Emily M. Lee

**Affiliations:** Division of Preclinical Innovation, National Center for Advancing Translational Sciences (NCATS), National Institutes of Health, Rockville, MD USA

**Keywords:** Neurological models, Tissue engineering, Assay systems, Neuroimmunology, Phenotypic screening

## Abstract

Microglia are the primary resident immune cells of the brain, playing both protective and deleterious roles in neurological diseases, which makes them an enticing therapeutic target. Here we developed a Neural Microglia Integrated Multicellular iPSC-derived Cultured Spheroids (NeuroMIMICS) system that enables measurements of both neural network activity and immune responses using human iPSC-derived microglia, astrocytes, and neurons. We validated microglia functionalities in these neural tri-cultures including phagocytic activity, directed motility, and inflammatory responses. RNAseq analysis revealed a phenotypical acquisition of immune functionality via microglia incorporation, supported by increased cytokine production in spheroids challenged with pathogen-like insults. We then demonstrated that the incorporation of healthy microglia corrected alpha-synuclein A53T-mediated dysfunctional phenotypes in the neural spheroids. This work demonstrates a unique immunocompetent functional neural model that is robust and suited for high-throughput screening, laying the groundwork for its application to accelerate the discovery of new therapeutics for neurological diseases.

## Abstract (web summary)

NeuroMIMICS, a high throughput, 3D human iPSC-derived neuron-astrocyte-microglia triculture spheroid model, captures neural activity and immune responses. Functional microglia enhanced immune signaling and rescued α-synuclein A53T deficits.

## Introduction

Microglia are the resident immune cells of the central nervous system (CNS) and are estimated to represent between 0.5 and 16.6% of all cells across different regions in the human brain, with distinct regional heterogeneity^1–3^. Microglia play critical roles in neurodevelopment via phagocytosis of apoptotic cells, synaptic pruning, and neurogenesis modulation, and continue to maintain CNS homeostasis post-neurodevelopment by surveying their surrounding microenvironment for pathogen-associated molecular patterns (PAMPs), damage-associated molecular patterns, or neurodegeneration-associated molecular patterns. Upon detection of an insult, healthy microglia engage in communication with surrounding cells, including neurons and astrocytes, migrate to the site of insult, mediate neuroinflammatory response via the release of chemokines and cytokines, and eliminate harmful substances through phagocytosis. However, in certain disease conditions, such as viral infection, Parkinson’s disease (PD), and amyotrophic lateral sclerosis, chronic microglial response has been known to exacerbate the disturbance of brain homeostasis, leading to more neuronal damage and worsening disease progression^4,5^. As evidence grows supporting the role of dysfunctional microglia in neurodegenerative diseases, there is an increased interest in targeting microglia as an alternative therapeutic approach for neurological disorders^4–6^.

Because human microglia are sufficiently distinct from rodent microglia, with significant transcriptomic and functional differences as well as differential expression of susceptibility genes associated with human neurological disorders, the use of human microglia is necessary for neurotherapeutic discovery and development^7–10^. The advances in the development of protocols to generate human induced-pluripotent stem cell (hiPSC) derived microglia^11–13^ have enabled the development of human-relevant microglia models that can be scaled up in two-dimensional (2D) culture for high-throughput screening (HTS) of thousands of compounds; however, environment-dependent microglial phenotypes are lost in 2D microglia monocultures^8,14^. The use of 2D co-culturing or tri-culturing with neurons and astrocytes can partially restore microglial in vivo signatures by increasing the cell type complexity and allowing direct interactions between cell types^10,15^. However, studies have shown that the three-dimensional (3D) matrix or tissue microenvironment is necessary for a more physiologically relevant microglia model that includes a diverse, multipolar phenotype population as found in vivo^8,14,16–18^. In recent years, several groups have successfully incorporated microglia into 3D human brain organoids (HBO) by either co-culturing mature microglia with HBOs, co-culturing microglial progenitor cells with HBOs, or by allowing spontaneous formation of innate microglia in HBOs (reviewed in ref. ^19^). Although human microglia-integrated organoids resemble the complex cellular architectures of the human brain^20–23^ and have been used to model several neurological disorders, such as Alzheimer’s disease and PD^12,20,24^, they are not suitable for HTS due to their low batch-to-batch reproducibility, inter-organoid heterogeneity, extensive culturing protocols, and technical difficulty in measuring functional neural activity. Given that 2D platforms may oversimplify the microenvironment required for an accurate microglial response, and HBOs introduce significant technical challenges that hinder drug screening campaigns, there is a pressing need for a predictable, human neural model with immune competency that can be used to support early drug discovery.

In this study, we developed protocols to incorporate microglia into functional brain region-specific neural spheroids to generate a 3D tri-culture platform that demonstrates both immunocompetency and functional neural activity profiles, referred to here as the neural microglia integrated multicellular iPSC-derived cultured spheroids (NeuroMIMICS) system. These NeuroMIMICS consist of customizable ratios of pre-differentiated human iPSC-derived neurons, astrocytes, and microglia seeded in 384-well plate format. By utilizing commercially available, validated, and cryopreserved human iPSC-derived cells, the NeuroMIMICS requires a shorter experimental period of maturation to display reproducible and measurable functional network activity. NeuroMIMICS also shows a wide range of microglial activities, including clearing of apoptotic cells, phagocytic activity, directed motility, and inflammatory responses to internal and external stimuli. Moreover, as proof of concept, we evaluated the functionality of healthy microglia in a disease-like NeuroMIMICS model and showed that the incorporation of healthy microglia can avert A53T alpha-synuclein-associated functional deficit reflected in neural network activity alteration measured in an HTS platform. Together, we showed that this physiologically relevant NeuroMIMICS platform enables pharmacological targeting of microglial functions and provides comprehensive readouts of neural activity as well as neuroimmune responses, positioning this platform as a promising tool for investigating potential therapeutics and advancing early drug discovery for neurological diseases.

## Results

### Generation of microglia-containing neural spheroids with controllable cell type combinations in a high-throughput platform

A major challenge for creating a functional NeuroMIMICS that incorporates neurons, astrocytes, and microglia is the compatibility of the cell culture conditions, which should not only ensure the survival of all three cell types but also support the functional activity of each cell type during relevant homeostasis and disease conditions. We previously reported the use of a BrainPhys-based media that supports synchronized neural network activity in neural spheroids; however, this media lacks critical supplements for the survival of microglia, highlighting the need to modify the media composition for the generation and maintenance of the tri-culture spheroids^25,26^. To optimize the media composition, we seeded glutamatergic neurons, astrocytes, and microglia together in 2D and tested a panel of microglial supplements (M-CSF, TGF-β2, and cholesterol) in BrainPhys media together with neuronal activity supportive supplements previously established in the designer brain-region specific neural spheroid platform (N2, B27, BDNF, GDNF, laminin, ascorbic acid, and cAMP)^26,27^. Tri-cultured cells maintained in different media for 21 days were evaluated for viability and morphology using cell type markers for neurons (MAP2), astrocytes (GFAP), and microglia (IBA1). While 100 ng/mL M-CSF, 2 ng/mL TGF-β2, and 1.5 µg/mL cholesterol were found to enhance microglia survival, we found that cAMP drastically suppressed the survival of microglia despite the addition of microglial supplements (Supplementary Fig. 1), which is in line with previous reports^22^. The final media composition of BrainPhys, N2, B27, BDNF, GDNF, laminin, ascorbic acid, TGF-β2, M-CSF, and cholesterol was used for further studies and referred to hereinafter as tri-culture media.

**Fig. 1:**
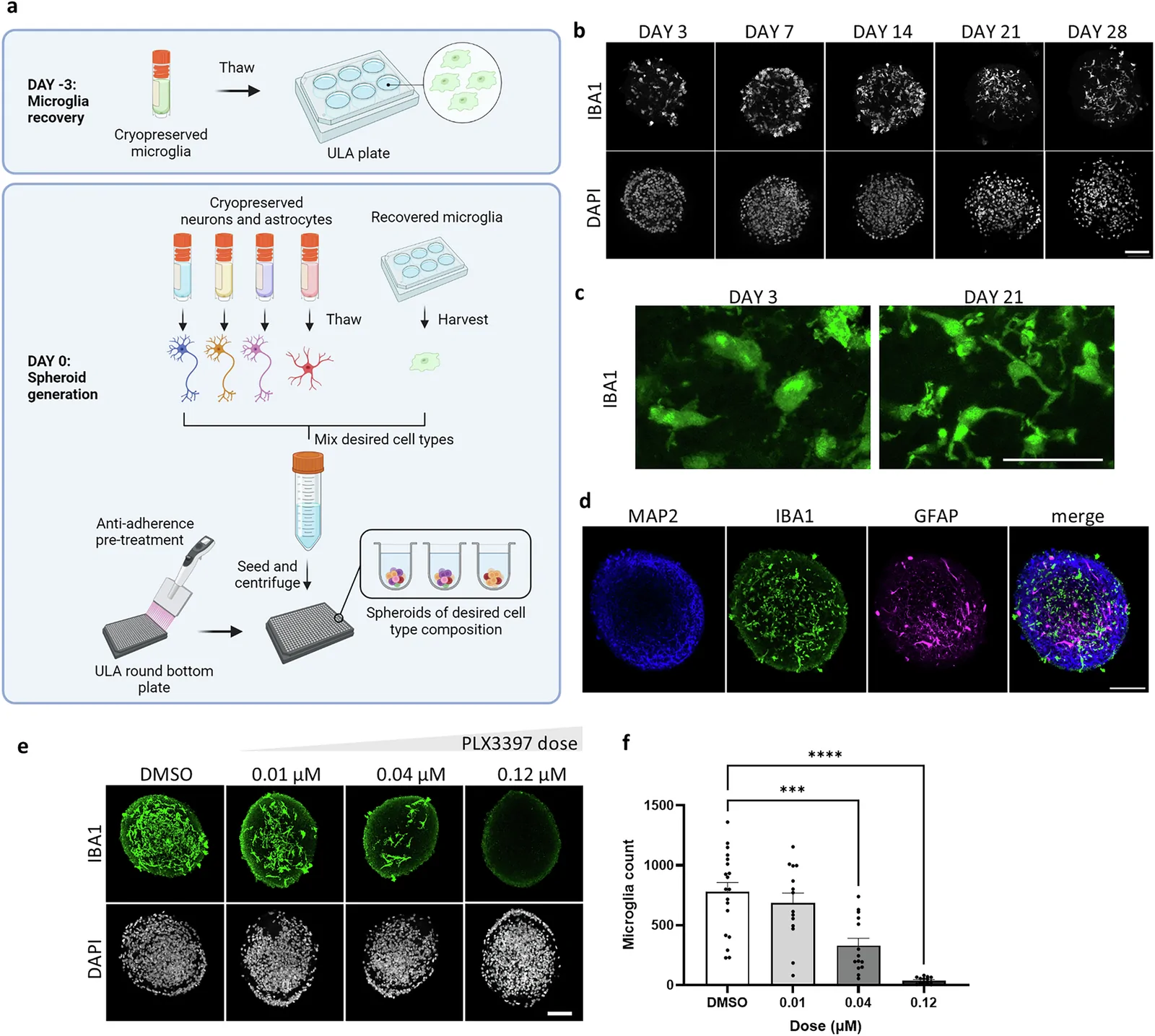
Generation of microglia, neurons, and astrocytes containing tri-culture spheroids in 384-well microplates. **a** Schematic of the microglia-containing tri-culture spheroids seeding protocol. **b** Confocal images (25×) of immunostaining for microglial marker IBA1 in tri-culture spheroids comprised of glutamatergic neurons, astrocytes, and microglia at 3, 7, 14, 21, and 28 days after seeding as spheroids to visualize microglia incorporation over time. Representative IBA1 and DAPI (nuclei) images are presented as maximum projections of 5.7 µm z-stacks captured approximately 60 µm into each spheroid. Scale bar = 100 µm. **c** Confocal images (25X) of IBA1-positive cells inside day 3 and day 21 tri-culture spheroids containing glutamatergic neurons, astrocytes, and microglia. Representative images are presented as maximum projections of 5.7 µm z-stacks. Scale bar = 50 µm. **d** Single plane confocal images of a representative tri-culture spheroid fixed at day 21 and immunostained for neuronal marker (MAP2), astrocyte marker (GFAP), and microglia marker (IBA1). Scale bar = 100 µm. **e** Confocal images (25×) of tri-culture spheroids after 96 h of treatment with DMSO or PLX3397 (final concentration of 0.01, 0.04, or 0.12 µM). Spheroids were immunostained for IBA1 and counterstained for DAPI. Representative images are presented as maximum projections of 25 µm z-stacks. Scale bar = 100 µm. **f** Quantification of IBA1+ cells in spheroids treated with DMSO or PLX3397 for 96 h. Statistical analysis was done using Welch’s one-way ANOVA followed by Dunnett’s multiple comparisons test. Data for each graph were collected from *n* = 3 independent experiments with 4–8 replicates in each experiment and presented as mean ± SEM. Error bars indicate the standard error of the mean. Asterisk symbols represent *P < 0.05, **P < 0.01, ***P < 0.001, ****P < 0.0001, and ns = not significant. “Figure 1. Generation of microglia, neurons, and astrocytes containing tri-culture spheroids in 384-well microplates.” Created in BioRender. Lee, E. (https://BioRender.com/im40vkb) is licensed under CC BY 4.0.

We next confirmed the generation of tri-culture spheroids in 384-well ultra-low attachment (ULA) round-bottom plates (Fig. 1a). Microglia were first thawed and seeded in 6-well ULA plates 3 days prior to spheroid generation (referred to as day −3) to promote microglia recovery from cryopreservation before incorporation into spheroids. On day 0, iPSC-derived, cryopreserved glutamatergic neurons and astrocytes were thawed, and microglia were harvested from 6-well plates and mixed in a 10:1:2 ratio (neuron:astrocyte:microglia), followed by seeding into 384-well ULA round-bottom plates at 13,000 cells/spheroid. We found that while the ULA treatment was sufficient to force the aggregation of neurons and astrocytes into spheroids, it was not always sufficient to prevent microglial attachment to the bottom of the plate, and we therefore incorporated an additional pre-treatment of anti-adherence rinsing solution to the microplate for the successful and reproducible integration of microglia into the spheroids (Supplementary Fig. 2a, bottom panel). This pre-treatment allowed for the incorporation of microglia into spheroids, which was dependent on initial input ratios (Supplementary Fig. 2b).

To track microglia integration, we tri-cultured neurons, astrocytes, and GFP-labeled microglia using the adjusted and optimized protocol (Fig. 1a). We observed a spontaneous aggregation of neurons, astrocytes, and microglia in each well within 24 h post-seeding, and by day 3, brightfield and GFP imaging revealed microglia were either inside the spheroid or adhered to the outside of the spheroid (Supplementary Fig. 3a, N+A+M panel). Interestingly, we observed that GFP-labeled microglia did not incorporate into neural spheroids as efficiently or homogeneously when astrocytes were not included (Supplementary Fig. 3a, N+M panel), as evident by a lower percentage of IBA1-positive microglia in neuron + microglia (N+M) spheroids compared to tri-culture neuron + astrocyte + microglia (N+A+M) spheroids (Supplementary Fig. 3b). To further examine the spatial positioning and morphology of the microglia in tri-culture spheroids over time, we performed immunofluorescent staining at 5 different time points (day 3, 7, 14, 21, and 28) and observed that microglia migrate into the center of the tri-culture spheroids over time and remain integrated until at least day 28 (Fig. 1b). Interestingly, microglia in day 3 tri-culture spheroids exhibit an ameboid morphology but further adapt a ramified morphology by day 21, this is suggestive of a dynamic switch from a motile and activated state early during tri-culture spheroid formation to a more homeostatic state in the later established spheroid tissue (Fig. 1b, c)^28^. This ramified morphology of microglia was evident in both neuron-microglia co-cultures and neuron-microglia-astrocyte tri-culture spheroids, which displayed more complex branching than those in 2D tri-culture, suggesting that the 3D microenvironment is preferable to drive a homeostatic microglia state (Supplementary Fig. 3c).

Our previous study determined that 21 days in culture is sufficient for neuron-astrocytes co-culture spheroids to establish synchronized network activities^26^, therefore, we selected day 21 as the endpoint for tri-culture spheroids, given our observations that microglia are both efficiently integrated and adopt a more physiologically relevant, homeostatic state-like morphology by day 21. Immunostaining of day 21 spheroids for the neuronal marker MAP2 and the astrocyte marker GFAP further demonstrated that both neurons and astrocytes were present with elongated processes, indicating the healthy maintenance of all three cell types in such tri-culture environments (Fig. 1d).

To demonstrate that microglia incorporated into tri-culture spheroids can be pharmacologically modulated, we treated microglia-containing tri-culture spheroids with pexidartinib (PLX3397), which is a compound that blocks colony-stimulating factor 1 (CSF1) receptor and results in microglia depletion, as seen both in vivo and in vitro systems^29,30^. Here, tri-culture spheroids were treated with PLX3397 at different doses (0 or DMSO vehicle control, 0.01, 0.04, and 0.12 µM) for 96 h before fixation and immunostaining against IBA1, MAP2, and GFAP. Using an automated high-content imaging system, we acquired confocal images of the spheroids, quantified the number of IBA1-positive cells, and calculated the total intensity of MAP2 and GFAP in each spheroid following compound treatment (Fig. 1e and Supplementary Fig. 4). We found a dose-dependent reduction in the number of microglia, while no significant alteration was found in the intensities of neuron and astrocyte markers (Fig. 1f and Supplementary Fig. 4), suggesting that the incorporated microglia can be depleted from tri-culture spheroids by PLX3397 in a dose-dependent manner, and such depletion was selective for microglia as predicted.

### Microglia in tri-culture spheroids respond to endogenous and exogenous stimuli, exhibit motility, and phagocytic activity

**Fig. 2:**
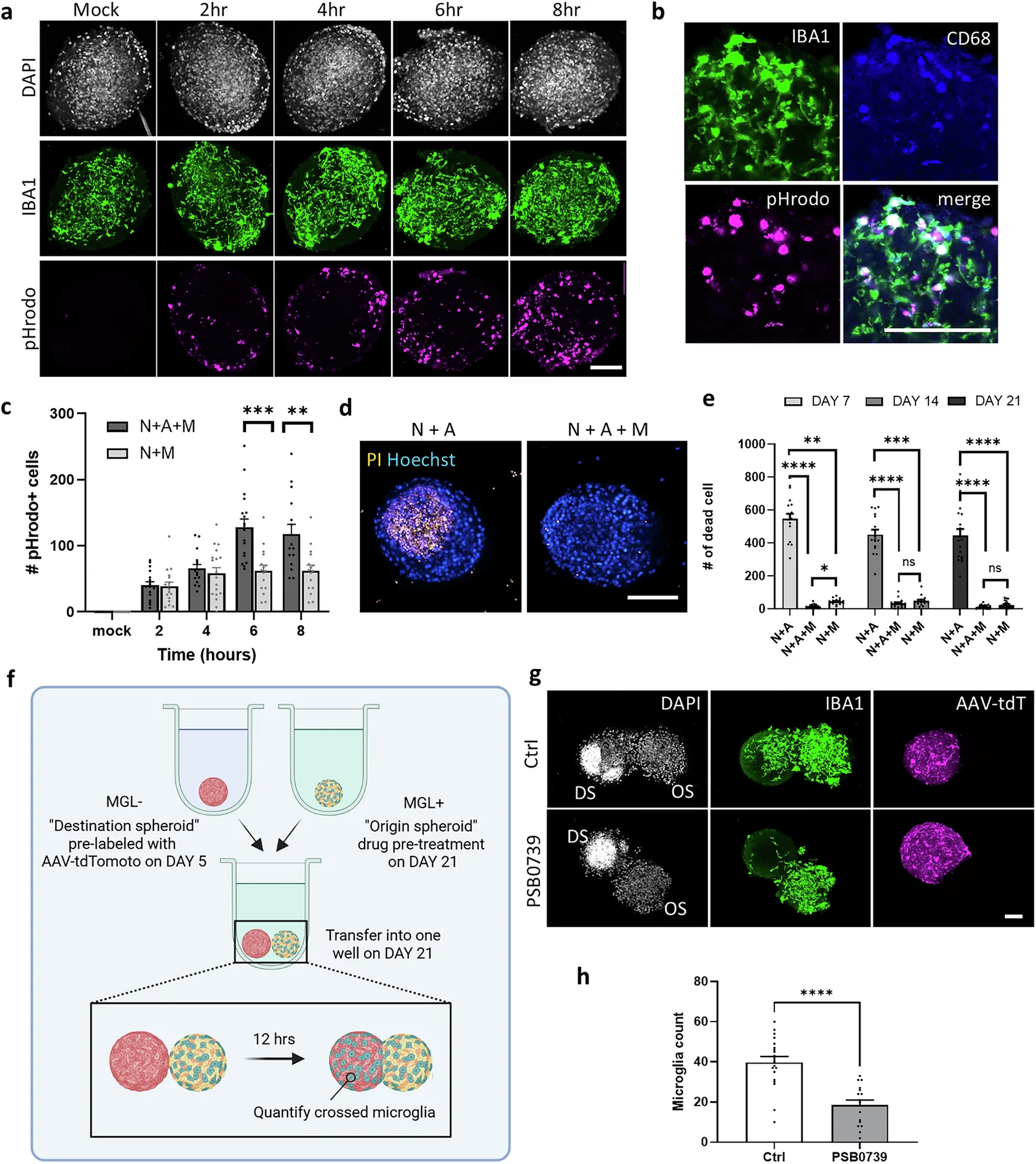
Microglia in tri-culture spheroids exhibit intact microglial functionalities. **a** Confocal images (25×) of day 21 tri-culture spheroids comprised of glutamatergic neurons, astrocytes, and microglia fixed after a 2-, 4-, 6-, or 8-h exposure to pHrodo bioparticles (100 µg/ml) or mock. Representative images are maximum projections of 150 µm z-stacks. **b** Single-plane confocal images (25×) acquired from a day 21 tri-culture spheroid exposed to pHrodo for 8 h, showing colocalization of IBA1, CD68, and pHrodo. **c** Quantification of pHrodo+ cells from neuron-microglia co-culture spheroids (N+M) and tri-culture spheroids (N+A+M) exposed to pHrodo or mock. Unpaired t-test with Welch’s correction was used. **d** Confocal images (20×) of day 21 spheroids with or without microglia following PI and Hoechst incubation. Representative images are maximum projections of 200 µm z-stacks. **e** Quantification of the number of PI-labeled dead cells in co-culture or tri-culture spheroids on day 7, 14, or 21. Batch-to-batch variability of dead-cell burden at seeding was estimated to be approximately CV = 5.2% for tri-culture spheroids (N+A+M), CV = 6.4% for co-culture (N+A) spheroids, and CV = 5.6% for co-culture (N+M) spheroids. Kruskal–Wallis test followed by Dunn’s multiple comparisons was used. **f** Schematic of the assembloid-migration assay used in (**g**, **h**). **g** Confocal images (10×) of assembloids treated with vehicle control or 10 µM PSB0739. **h** Quantification of IBA1+ cells residing in “destination spheroids” after vehicle control or PSB0739. Unpaired t-test was used. Data for each graph were collected from *n* = 3 independent experiments with 4–8 replicates in each experiment, and presented as mean ± SEM. Error bars indicate the standard error of the mean. Asterisk symbols represent *P < 0.05, **P < 0.01, ***P < 0.001, ****P < 0.0001, and ns = not significant. Scale bar = 100 µm. “Figure 2. Schematic of the assembloid-migration assay,” created in BioRender. Lee, E. (https://BioRender.com/0jtdzwp) is licensed under CC BY 4.0.

Following the activation by external stimuli, microglia play a critical role in maintaining brain homeostasis via their phagocytic functions. To assess whether microglia in tri-culture spheroids have phagocytic activities, we challenged microglia-containing tri-culture or microglia-absent co-culture spheroids with pHrodo red *S. aureus* bioparticles (100 µg/ml) which become highly fluorescent in low pH environments, such as acidifying endosomes and lysosomes for 2, 4, 6, or 8 h (Fig. 2a and Supplementary Fig. 5). In microglia-containing tri-culture spheroids, we observed a time-dependent increase in pHrodo signal, whereas no significant increase over background signal was detected in microglia absent co-culture spheroids (Fig. 2c and Supplementary Fig. 5b). Co-immunostaining of IBA1 and CD68 on tri-culture spheroids treated with pHrodo for 8 h further demonstrated that the detected pHrodo signal colocalized with IBA1 and CD68, confirming the involvement of microglial phagocytic activity associated with the observed pHrodo-labeling (Fig. 2b). Specifically, we found that pHrodo uptake began from the outer layers and gradually appeared in the center of the spheroids after 8 h of incubation with pHrodo-bioparticles, which is likely a result of slow penetration of the bioparticle into the densely packed 3D tissue (Fig. 2a). Together, the co-localization of microglia marker IBA1 with pHrodo (Fig. 2b), alongside the lack of co-localization with GFAP as well as the absence of pHrodo signal in neuron-astrocyte co-culture or neuron monoculture spheroids (Supplementary Fig. 5b, c), suggests that observed phagocytic capacity in the tri-culture spheroids is due to the presence of microglia.

Cryopreserved neurons require a dead-cell removal step to minimize non-viable neurons in spheroids^26^. To confirm whether microglia can remove apoptotic cells in tri-culture spheroids, we formed co-culture or tri-culture spheroids without such dead-cell removal step and examined the viability of each spheroid on day 7, 14, and 21 using propidium iodide (PI) dye which selectively labels dead cells in live spheroids (Fig. 2d). We found that both tri-culture spheroids (N+A+M) and neuron-microglia co-culture spheroids (N+M) contain significantly fewer numbers of dead cells compared to neural spheroids without microglia (N+A) in all three time points examined, suggesting that dead cells were effectively cleared from microglia-present spheroids within 7 days in culture (Fig. 2d, e). Given that microglia in tri-culture spheroids can sufficiently detect and remove dead cells, we proposed that such signaling in microglia-absent spheroids could be used as a trigger for directed motility evaluation. We therefore designed an assembloid-migration assay using two different sets of 21-day old spheroids (Fig. 2f), where “origin spheroids” were microglia-containing (MGL+) tri-culture spheroids generated using glutamatergic neurons, astrocytes, and microglia, while “destination spheroids” were microglia-absent (MGL−) co-culture spheroids contained only glutamatergic neurons and astrocytes. To distinguish between origin spheroids and destination spheroids, we transduced destination spheroids with a control AAV at day 5 to express the red fluorescent protein tdTomato. Tri-culture origin spheroids and destination spheroids were then paired into a co-culture well to allow the formation of an assembloid. To establish the timeframe for the assembloid-microglia migration assay, we conducted a preliminary trial using GFP-labeled microglia and live imaging over the course of 18 h (Supplementary Fig. 6), and observed that within 12 h, GFP-labeled microglia in tdTomato-absent origin spheroids migrated toward tdTomato-positive destination spheroids without posing major challenges for 3D quantification.

Based on these results, we hypothesized that ADP/ATP released by dead cells in MGL- spheroids acted as a trigger for microglial-directed motility in assembloids. To test this hypothesis, we treated the origin spheroid with PSB0739, an inhibitor of purinergic receptor P2RY12, which has been previously shown to efficiently suppress microglial motility both in vivo and in vitro 2D cultures^31–33^. We found significantly fewer IBA1+ cells migrated into the destination spheroids in 12 h following PSB0739 treatment, compared to the mock control group (Fig. 2g, h). These results indicate that microglia in the tri-culture spheroids display P2RY12-dependent directed motility. Together, these data show that microglia in the tri-culture spheroids platform retain physiologically relevant functions, such as stimuli-driven migration and phagocytosis that are necessary for the regulation and maintenance of CNS homeostasis.

### Microglia-integrated neural spheroids exhibit cell-type composition-dependent spontaneous calcium oscillation

Following the successful tri-culture of hiPSC-derived glutamatergic neurons, astrocytes, and microglia in a 3D spheroid platform, we sought to evaluate whether these tri-culture neural spheroids exhibited synchronized network activity that is dependent on neuronal cell-type compositions. Previously, we have shown that neural spheroids cultured in a BrainPhys-based media without microglia develop reproducible and spontaneous synchronized network activity after 21 days in culture, which can be captured in a high-throughput fashion by recording calcium oscillations in single-well resolution with 384 wells captured simultaneously per plate, and we have shown that this activity is dependent on neuronal composition^26^. Here, with the addition of microglia and the necessary modifications made in culturing media composition, we investigated whether the tri-culture spheroids retain high-throughput compatible functional readout, and whether the incorporated microglia influence spontaneous network activity. To do this, we utilized the assembly protocol and tri-culture media described above to assemble a panel of neural spheroids of varying neuronal type compositions (Table 1), including glutamatergic or dopaminergic spheroids as mono-cultures (Gl or D), co-cultured with astrocytes (A) or microglia (M), or as tri-cultures with astrocytes and microglia; as well as microglia-containing brain region-specific spheroids mimicking the prefrontal cortex (PFC) or ventral tegmental area (VTA)^26^. To validate cell type presence on day 21 in each spheroid, we performed whole tissue immunostaining of cell type markers and confirmed that all neuronal cell types tested in this study are compatible with the optimized protocol (Supplementary Fig. 7).

**Table 1:** Seeding cell numbers per spheroid

Spheroid type	# Glutamatergic neurons/spheroid	# Dopaminergic neurons/spheroid	# GABAergic neurons/spheroid	# Astrocytes/spheroid	# Microglia/spheroid
Gl	10,000	N/A	N/A	N/A	N/A
Gl+A	10,000	N/A	N/A	1000	N/A
Gl+A+M	10,000	N/A	N/A	1000	2000
Gl+M	10,000	N/A	N/A	N/A	2000
D	N/A	10,000	N/A	N/A	N/A
D+A	N/A	10,000	N/A	1000	N/A
D+A+M	N/A	10,000	N/A	1000	2000
D+M	N/A	10,000	N/A	N/A	2000
VTA (MGL−)	500	6500	3000	1000	N/A
VTA (MGL+)	500	6500	3000	1000	2000
PFC (MGL−)	7000	N/A	3000	1000	N/A
PFC (MGL+)	7000	N/A	3000	1000	2000

**Fig. 3:**
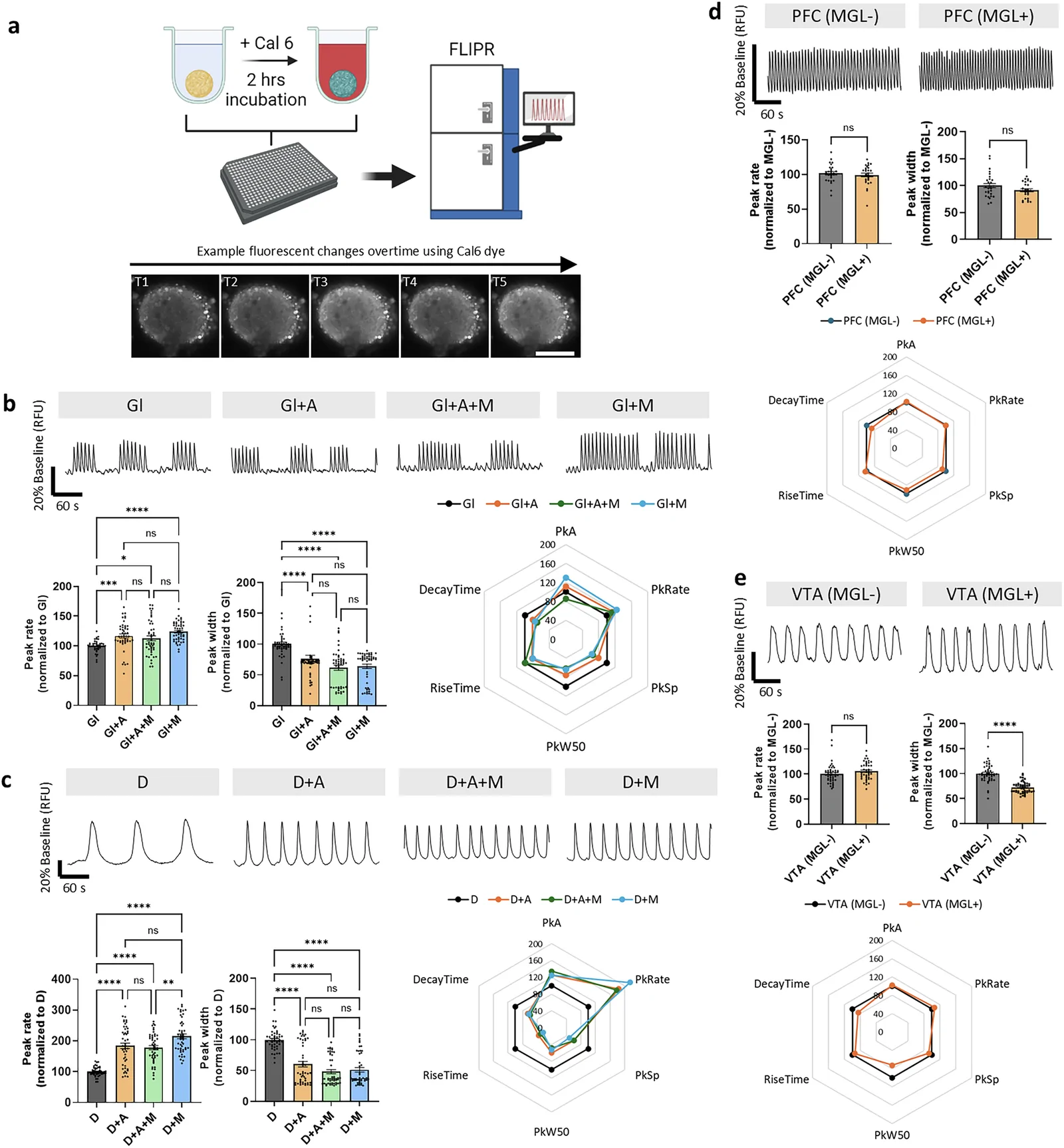
Microglia-integrated neural spheroids exhibit cell-type composition-dependent spontaneous calcium oscillation. **a** Schematic of calcium oscillation detection in neural spheroids using Cal6-FLIPR assay (top) and representative single-plane live imaging of a tri-culture spheroid after 2 h of incubation in Cal6 dye using a Phenix confocal (10×) with 2.5 s intervals (bottom). Scale bar = 100 µm. **b** Representative spontaneous calcium oscillations (top) and quantification of peak parameters (bottom) from glutamatergic (Gl) monoculture spheroids, co-culture spheroids with astrocytes (A) or microglia (M), and tri-culture spheroids. **c** Representative spontaneous calcium oscillations (top) and quantification of peak parameters (bottom) from dopaminergic (D) monoculture, co-culture, and tri-culture spheroids. **d** Representative spontaneous calcium oscillations (top) and quantification of peak parameters (bottom) from PFC-like spheroids with or without microglia (MGL). **e** Representative spontaneous calcium oscillations (top) and quantification of peak parameters (bottom) from VTA-like spheroids with or without MGL. Column graphs show quantifications of peak rate and peak width using data collected from *n* = 3 independent experiments and 16 replicates in each experiment, and presented as mean ± SEM. Error bars indicate the standard error of the mean. Welch’s ANOVA test followed by Dunnett’s multiple comparison was used for peak rate in (**b**, **c**). Kruskal–Wallis test followed by Dunn’s multiple comparisons tests was used for peak width in (**b**, **c**). Mann–Whitney tests were used in (**d**, **e**). Asterisk symbol represents *P < 0.05, **P < 0.01, ***P < 0.001, ****P < 0.0001, and ns =  not significant. Radar plots demonstrate the alterations of six key peak parameters within each family of spheroids using the mean of each type of spheroid normalized to Gl only, D only, or PFC or VTA brain-region-like spheroids without microglia. “Figure 3. Schematic of calcium oscillation detection in neural spheroids,” created in BioRender. Lee, E. (https://BioRender.com/006edwz) is licensed under CC BY 4.0.

We measured spontaneous calcium oscillations in spheroids of different cell-type compositions using the “FLIPR” penta high-throughput cellular screening system, which is a whole plate reader equipped with a high speed, high sensitivity EMCCD camera for fluorescent detection (Fig. 3a). We found that monoculture spheroids containing mostly glutamatergic neurons (Gl) exhibit a burst activity pattern that varies between wells (Supplementary Fig. 8). While the further incorporation of astrocytes (Gl+A), microglia (Gl+M), or both (Gl+A+M) alters the oscillation pattern from monoculture glutamatergic spheroids, the bursting phenotype remains (Fig. 3b), making the interpretation of statistical analysis challenging (Supplementary Table 1). In contrast, monoculture, co-culture, or tri-culture spheroids with mostly dopaminergic neurons and brain region-specific spheroids exhibit steady oscillation patterns with high reproducibility between wells (Fig. 3c and Supplementary Table 1). Specifically, we found that when compared to dopaminergic only spheroids (D), the incorporation of astrocytes (D+A) or microglia (D+M) significantly alters the calcium oscillation patterns, including increased peak rate and reduced peak width (Fig. 3c). However, the calcium oscillation of dopaminergic tri-culture spheroids (D+A+M) differed slightly from D+M co-cultured spheroids in peak rate but not peak width, while not significantly different from D+A co-culture spheroids (Fig. 3c).

When further looking at spheroids with more complex neuronal cell-type compositions, we found that for both PFC-like and VTA-like spheroids, the incorporation of microglia did not significantly alter most peak parameters, such as peak rate; however, it significantly reduces the peak width of VTA-like spheroids but not PFC-like spheroids (Fig. 3d, e). Together, these results suggest that the spheroid generation protocol and culturing conditions developed in this study support the assessment of neural network electrophysiological properties, which can be used as a high-throughput functional readout, and such neural activity is sensitive to the cell-type composition of the spheroid.

### Tri-culture spheroids have transcriptomic profiles suggesting enhanced neuroimmune functionality and synaptic pruning via microglia-incorporation

**Fig. 4:**
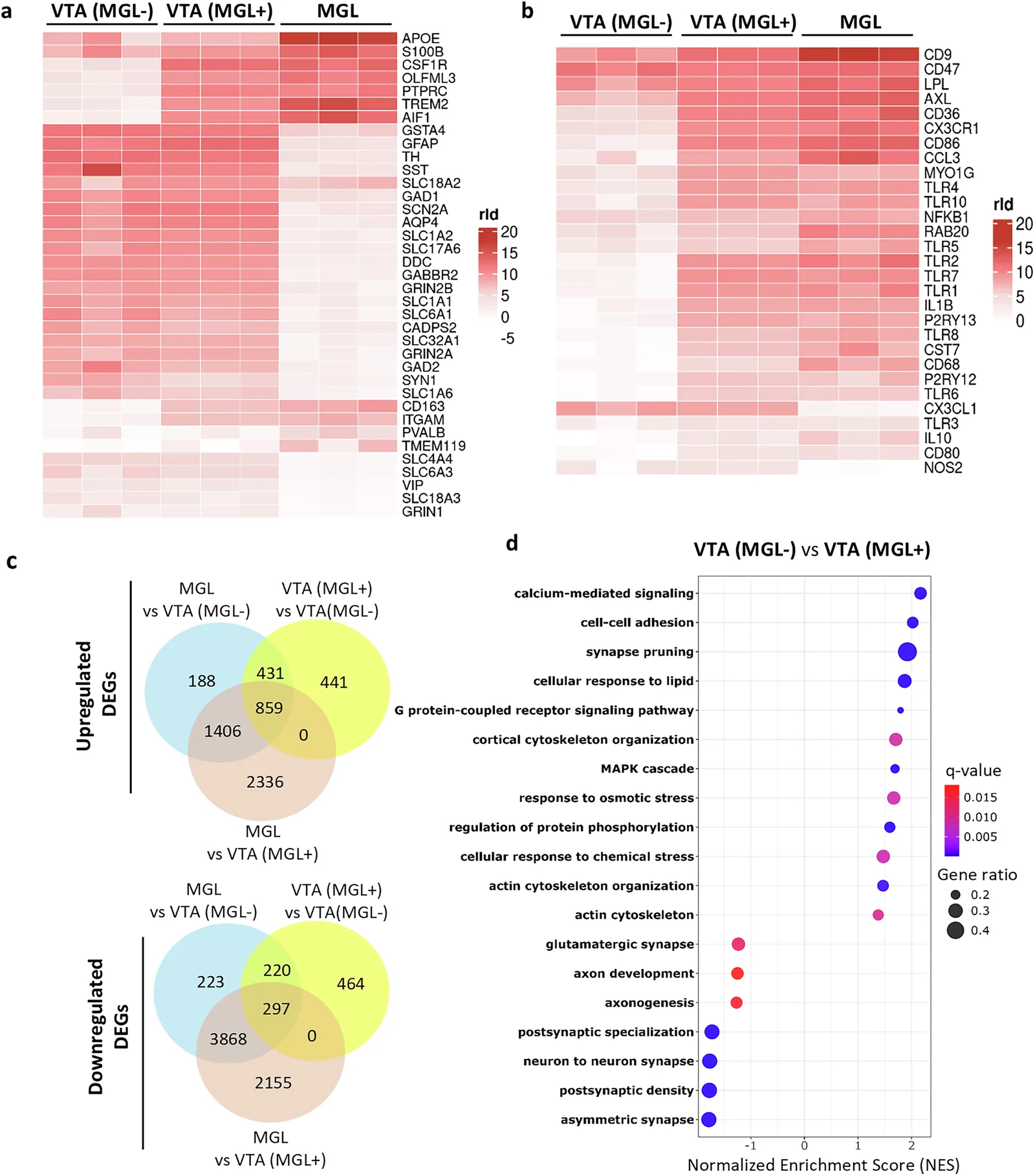
Transcriptomic profiles of VTA-like tri-culture spheroids. **a** Heatmap showing the expression levels of genes associated with cell type identities in VTA-like spheroids without microglia (MGL−), with microglia (MGL+), or microglia monocultures (MGL). **b** Heatmap of genes associated with immune functionalities in VTA-like spheroids with (MGL+) or without microglia (MGL-) and in microglia monocultures (MGL). **c** Venn diagrams showing significant up- or downregulated differentially expressed genes (DEGs) between the comparative cultures, using a threshold of log2 fold change >1 and *p* < 0.05 significance. **d** Selected set of gene set enrichment analysis (GSEA) identified pathways associated with genes that are upregulated or downregulated in VTA (MGL+) compared to VTA (MGL−).

To characterize and gain insight into the microglia-integrated tri-culture spheroids, we probed transcriptomic changes related to the microglia integration in neural spheroids. For this, we performed bulk RNA sequencing comparing day 21 spheroids with or without microglia and microglia-only cultures. For the rest of the studies, we chose to focus on VTA-like spheroids, given their inclusion of both dopaminergic and glutamatergic neurons, as well as physiological relevance to a wide range of high-impact diseases, such as PD, which affects approximately 1% of individuals over 60 worldwide^34^. We compared the gene expression profiles of three groups: VTA-like spheroids without microglia (MGL−), VTA-like spheroids with microglia (MGL+) and microglia only (MGL), using a threshold of log2 fold change >1 and *P* < 0.05 significance. MGL only cultures expressed expected microglia-specific marker genes, such as *CSF1R, OLFML3, TREM2, AIF1, CD163, PTPRC, ITGAM*, and *TMEM119*. VTA-like spheroids without microglia (MGL−) expressed markers for glutamatergic (*SLC17A6, GRIN2B*, and *GRIN1*), dopaminergic (*TH, SLC18A2, SCNA2, DDC*, and *SLC6A3*), GABAergic neurons (*SST, GAD1, GABBR2*, and *GAD2*), as well as astrocytes (*GFAP, GSTA4, AQP4, SLC1A2*, and *SLC4A4*), while VTA (MGL+) expressed comparable levels of markers found in both MGL and VTA (MGL−) cultures, confirming the sustained presence of all cell types (Fig. 4a). Stability of cell populations were further confirmed using MUlti-Subject SIngle Cell deconvolution (MuSiC)^35,36^ (Supplementary Fig. 9a), and a panel of eleven genes of varying expression profiles (*APOE, C1QB, GAD2, GFAP, ITGAM, P2RY12, SLC1A6, SYNGAP, TLR2, TLR3*, and *TREM2*) validated using qRT-PCR (Supplementary Fig. 9b). Furthermore, we observed retention of microglia-associated neuroimmune capabilities in VTA (MGL+) spheroids as they had significant gene expression of genes associated with phagocytosis (*CD68, RAB20, HCK*, and *AXL*)^23,37–40^, directed motility (*P2RY12, HCK*)^39,41^, inflammatory responses (*CD36, CD80, CD86, CCL3, NFKB1, RAB20, MYO1G, IL1B*, and pathogen recognition receptors *TLR1, TLR2, TLR3, TLR4, TRL5, TLR6, TLR7, TLR8*, and *TLR10*)^42–45^, as well as genes associated with crosstalk between microglia and other neural cell types (*CX3CR1, CX3CL1*, and *IL10*) (Fig. 4b).

As expected, given the different cell type compositions, we observed a large number of significant differentially expressed genes (DEGs) between the comparative cultures: 7492 DEGs in VTA (MGL−) vs. MGL, 2712 DEGs in VTA (MGL−) vs. VTA (MGL+) and 10,921 DEGs in VTA (MGL+) vs. MGL (log_2_fc ±1.0, *p* < 0.05; Fig. 4c). Broadly, we observed major transcriptomic patterns of gene expression changes within the groups that highlight cellular processes conferred by the microglia presence and incorporation (Fig. 4b), with significant increases in gene expression related to the innate immune system and processes like toll-like receptor (TLR) response (*TLR4, TLR2, TLR6*, and *TLR7*) in VTA (MGL+) spheroids vs. VTA (MGL−) spheroids. While some transcriptomic shifts may be due to the presence of different cell populations, we also observed changes that appeared to be independent of cell-type ratios as the only contributing factor. For example, we observed a significant downregulation in inflammatory microglia genes between MGL and VTA (MGL+) that may be related to microglia activation state in a pure population *vs* in a more complex brain-like environment (Fig. 4a). In addition, we performed gene set enrichment analysis to reveal functional changes to biological processes and molecular function pathways between VTA (MGL−) or VTA (MGL+) spheroids. To investigate DEGs independent of cell type specific expression, we first focused on a gene set of 1010 DEGs that were unique to VTA (MGL+) spheroids, by excluding 2112 genes from the DEGs of VTA (MGL−) vs. VTA (MGL+) that overlap with VTA (MGL−) vs. MGL DEGs, in order to capture functional changes apart from microglia-specific functionalities (Supplementary Fig. 9c). Interestingly, while we only observed upregulation of chromosome segregation pathway in VTA (MGL+) compared to VTA (MGL−), we observed 15 downregulated pathways associated with metabolic processes (Supplementary Fig. 9d). As most energy in the CNS is used for information processing which relies on the generation of action potentials, a reduction in ATP synthesis related pathways could indicate a refined neural network in the presence of microglia that is more energy efficient^46,47^. To explore this hypothesis, we probed whether the incorporation of microglia into VTA-like spheroids affects synaptic functionalities, and we found that the incorporation of microglia led to an upregulation of genes associated with synapse pruning, indicating a key functional role of microglia is maintained in the VTA (MGL+) spheroids (Fig. 4d and Supplementary Fig. 10)^24^. Correspondingly, we also observed a downregulation of genes associated with synaptic functions, including glutamatergic synapse, postsynaptic specialization, neuron to neuron synapse, postsynaptic density, asymmetric synapse, and axon development in the tri-cultured VTA (MGL+) spheroids (Fig. 4d and Supplementary Fig. 10). These findings support that microglia not only impart neuroimmune functionality to the tri-culture spheroids, but the incorporated microglia may also play a role in refining synaptic connections in tri-culture spheroids, consistent with findings from brain organoid studies^24^.

### VTA-like tri-culture spheroids produce inflammatory factors in response to exogenous stimuli

**Fig. 5:**
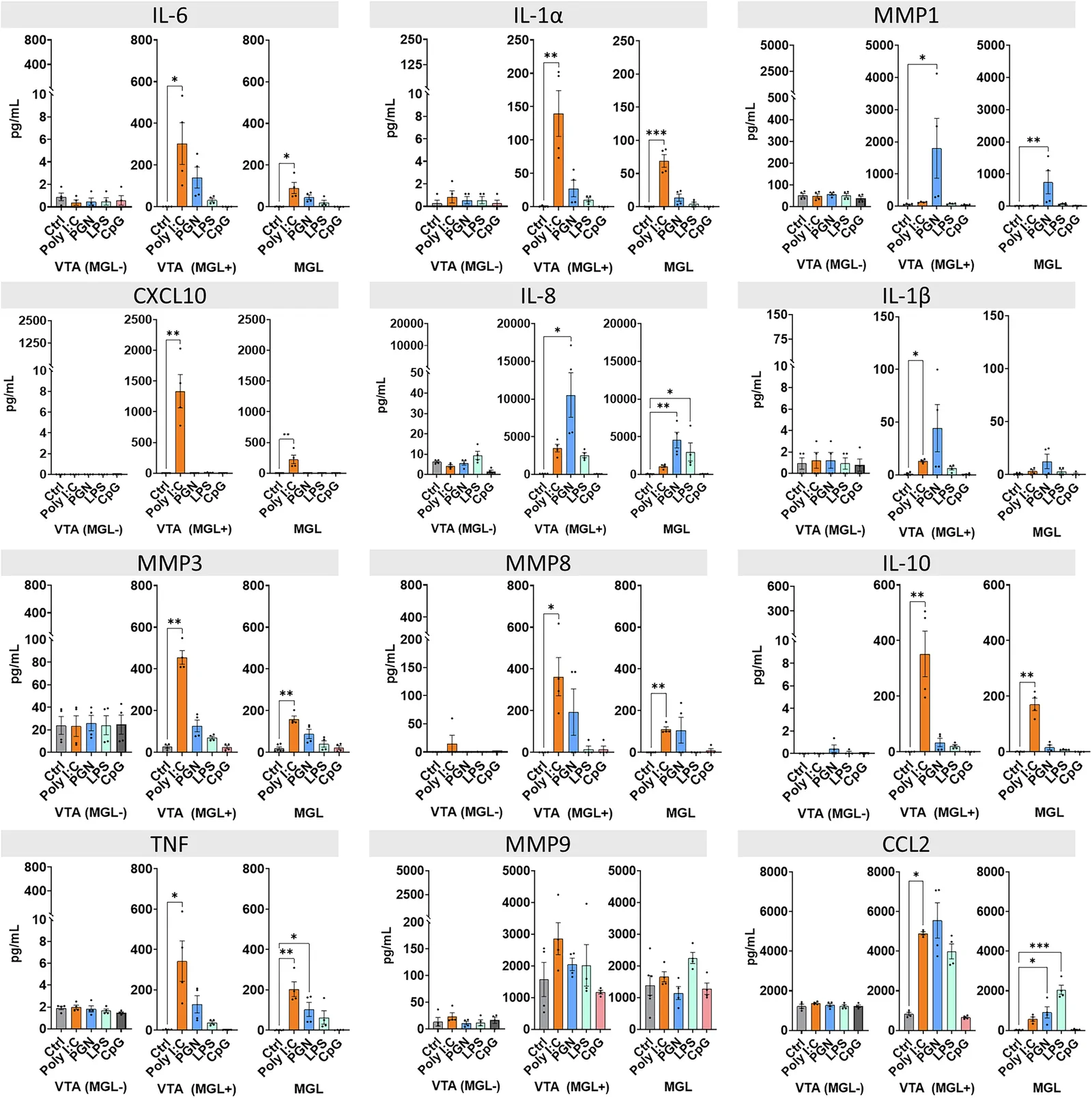
Cytokine profiles of VTA-like tri-culture spheroids. Cytokine levels detected in the supernatant of VTA-like spheroids with or without microglia (MGL) and microglia-only samples after stimulated for 24 h by polyinosinic:polycytidylic acid (poly I:C, 10 µg/mL), peptidoglycan (PGN, 10 µg/mL), lipopolysaccharide (LPS, 10 µg/mL), or cytosine-phosphate-guanine (CpG, 5 µM) using Luminex Multiplex Assay. Data were generated from two independent experiments (reported as mean ± SEM, error bars indicate the standard error of the mean), in which supernatants from 3 spheroids were pooled as 1 sample; *n* = 2 samples were collected from each independent experiment. Kruskal–Wallis test followed by Dunn’s multiple comparisons test was done for statistical analysis. Asterisk symbols represent *P < 0.05, **P < 0.01, ***P < 0.001, and ****P < 0.0001.

Activated microglia can produce both cytotoxic and neurotrophic factors in response to injury, ischemia and infection in the CNS^48^. Like other macrophage-like cells, microglia are known to express a wide range of TLR family members that recognize PAMPs and initiate innate immune responses^49^. Following our transcriptomic profiling, which confirmed TLR expression in VTA-like tri-culture spheroids (Fig. 4b), we further investigated whether these VTA-like tri-culture spheroids secrete cytokines upon such challenges. We measured a panel of cytokines and chemokines in spheroid supernatant 24 h after exposure to a panel of TLR agonists, including LPS (TLR4 agonist), poly I:C (TLR3 agonist), and peptidoglycan (PGN) from *S. aureus* (TLR2 agonist). As TLR9 expression was not detected in either MGL-only or VTA (MGL+) tri-culture (Fig. 4b), we also selected a class C cytosine-phosphorothioate-guanosine (CpG) oligonucleotide (TLR9 agonist) as a negative control. Without stimulation (CTRL), only MMP3, MMP9, and CCL2 were detected in the supernatants (Fig. 5). Specifically, baseline MMP3 was detected at low levels in all three spheroid types, while baseline MMP9 secretion was associated only with microglial presence, and baseline levels of CCL2 were not detected in unstimulated MGL-only cultures.

Poly I:C, PGN, or LPS treatments triggered distinct secretion profiles in microglia-containing VTA spheroids and microglia-only spheroids, whereas none of the 12 cytokines or chemokines measured were elevated in any stimuli treated VTA (MGL−) spheroids (Fig. 5). Specifically, compared to unstimulated samples, poly I:C led to increased production of IL-6 (*p* = 0.0341), IL-1α (*p* = 0.0039), CXCL10 (*p* = 0.0036), IL-1β (*p* = 0.0252), MMP3 (*p* = 0.0049), MMP8 (*p* = 0.0183), IL-10 (*p* = 0.0045), TNF (*p* = 0.0217), and CCL2 (*p* = 0.0483) in VTA (MGL+) spheroids. The production of these cytokines and chemokines was also detected in MGL-only samples following poly I:C treatment, but all at lower levels compared to VTA (MGL+) spheroids. Compared to unstimulated VTA (MGL+), PGN treatment induced detectable secretion of IL-6 (*p* = 0.1255, ns), IL-1α (*p* = 0.2025, ns), MMP1 (*p* = 0.0136), IL-8 (*p* = 0.0165), IL-1β (*p* = 0.0509, ns), MMP3 (*p* = 0.1448, ns), MMP8 (*p* = 0.4206, ns), IL-10 (*p* = 0.2817, ns), and TNF (*p* = 0.2077, ns) in VTA (MGL+) spheroids, and similarly, the increased levels of these cytokines and chemokines were also observed in PGN treated MGL-only samples compared to untreated, but less robust compare to VTA (MGL+). LPS induced increase in CCL2 production (*p* = 0.606, ns) in VTA (MGL+) spheroids compared to untreated controls, and significant increases in CCL2 (*p* = 0.0005) and IL-8 (*p* = 0.0168) in MGL only samples. While not statistically significant, we also detected elevated levels of IL-6, IL-1α, IL-8, IL-1β, MMP3, IL-10, and TNF in LPS- treated VTA (MGL+) and MGL-only samples. CpG did not significantly alter the level of any cytokine in this study, which is consistent with the absence of TLR9 expression evaluated using RNAseq (Fig. 4b). Together, we showed that microglia-integrated VTA-like spheroids respond distinctly toward different insults, indicating recruitment of different signaling pathways responsible for neuroimmune responses.

### Microglia exhibit neural protective roles against calcium activity changes associated with A53T alpha-synuclein expression in VTA-like spheroids

**Fig. 6:**
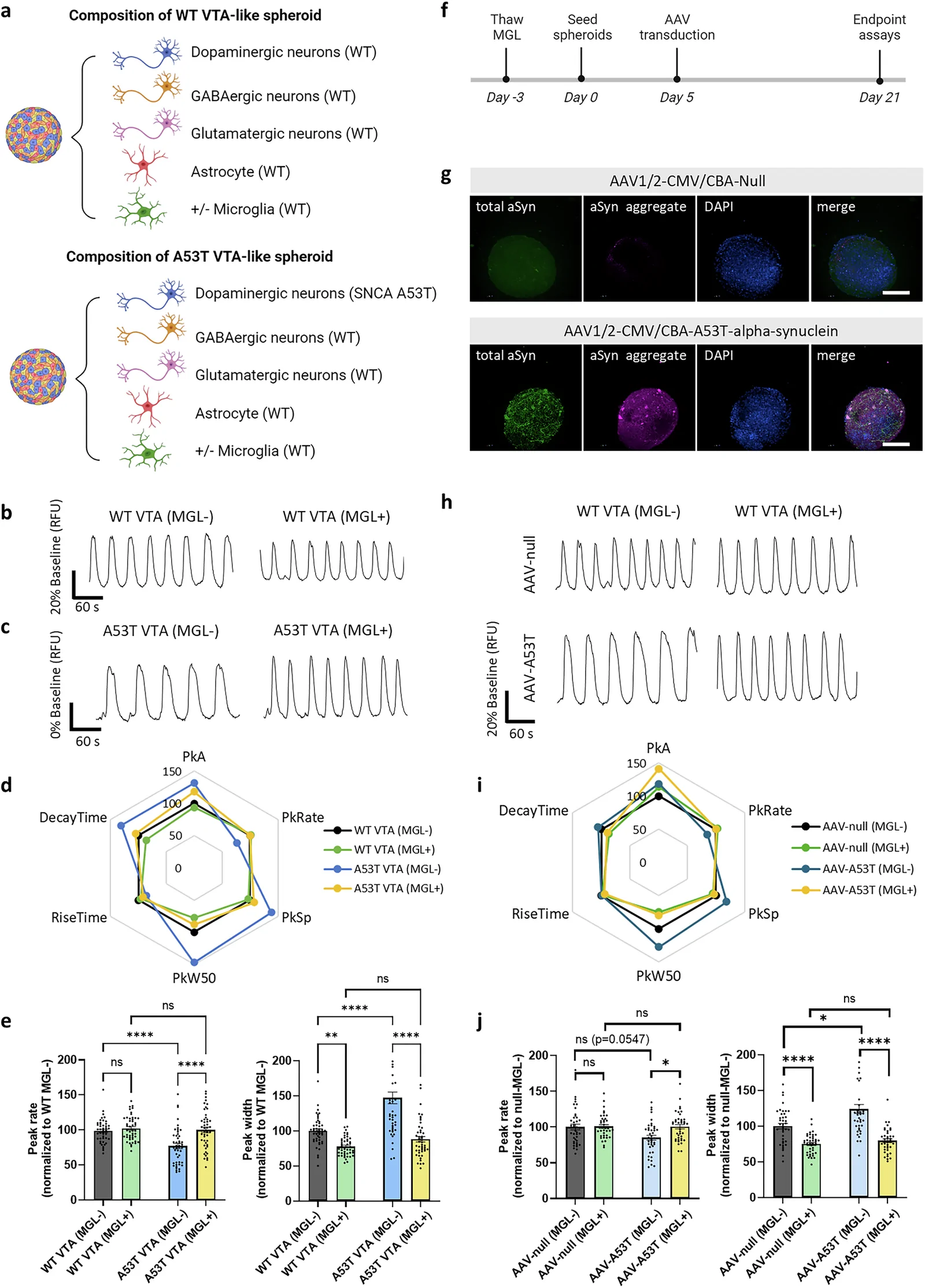
Microglia exhibit neuroprotective roles against calcium activity changes associated with A53T alpha-synuclein in VTA-like spheroids. **a** Schematic showing the cell-type compositions of WT VTA-like spheroids and A53T VTA-like spheroids used in this experiment. **b** Representative traces of spontaneous calcium oscillations in WT VTA-like spheroids with or without microglia (MGL±). **c** Representative traces of spontaneous calcium oscillations in A53T VTA-like spheroids with or without microglia (MGL±). **d** Radar plots of the multiparameter differences comparing WT and A53T VTA spheroids with or without microglia, using WT VTA as baseline control. **e** Quantification of peak rate and peak width using data collected from three independent experiments and 16 replicates in each experiment, and presented as mean ± SEM. **f** Schematic showing the timeline for AAV-mediated A53T-alpha-synuclein expression in VTA-like spheroids. **g** Representative images (20×) showing immunofluorescent staining of total alpha-synuclein and alpha-synuclein aggregates in WT VTA (MGL−) spheroids transduced with either a control AAV (top) or AAV-A53T-alpha-synuclein (bottom). Scale bar = 200 µm. **h** Representative traces of spontaneous calcium oscillations in WT VTA-like spheroids with or without microglia (MGL) that have been transduced with AAV-null or AAV-A53T-alpha-synuclein. **i** Radar plots of the multiparameter measurements comparing WT VTA spheroids with or without microglia transduced with control or A53T-alpha-synuclein overexpressing AAV, using WT VTA (MGL−) with control AAV as baseline control. **j** Quantification of peak rate and peak width using data collected from *n* = 3 independent experiments and 16 replicates in each experiment, and presented as mean ± SEM. Error bars indicate the standard error of the mean. Two-way aligned rank transform (ART) ANOVA followed by Tukey’s multiple comparisons test was used. Asterisk symbol represents *P < 0.05, **P < 0.01, ***P < 0.001, ****P < 0.0001, and ns = not significant. “Figure 6. Schematic showing the cell-type compositions of WT VTA-like spheroids and A53T VTA-like spheroids,” created in BioRender. Lee, E. (https://BioRender.com/54dwu1m) is licensed under CC BY 4.0.

As microglia are known to have a neuroprotective role in certain disease conditions^50^, we hypothesized that the incorporation of healthy microglia may influence disease-associated phenotypic alteration in calcium oscillation. To test this hypothesis, we compared the spontaneous calcium oscillation patterns between healthy wildtype (WT) VTA-like spheroids and VTA-like spheroids modeling PD, with or without microglia. As described before, WT VTA-like spheroids lacking microglia (“WT VTA (MGL−)”) were generated by mixing iPSC-derived dopaminergic, GABAergic, glutamatergic neurons, and astrocytes from a healthy donor. Tri-culture WT VTA-like (MGL+) spheroids were made with the addition of healthy iPSC-derived microglia (Fig. 6a). To generate PD-associated VTA-like spheroids (“A53T VTA”), WT dopaminergic neurons in both MGL+ and MGL− VTA-like spheroids were replaced with dopaminergic neurons differentiated from an isogenic iPSC line containing the heterozygous *SNCA* A53T variant (Fig. 6a). After 21 days in culture, there was no significant difference in spheroid viability between WT and A53T VTA (Supplementary Fig. 11a), suggesting that the genetic differences between the WT and *SNCA* A53T dopaminergic neuronal cell lines did not cause significant differences in the general health of the cells in the spheroids and therefore any changes in calcium oscillations are likely caused by effects of the mutation on network activity.

We next assessed calcium oscillations of WT and A53T VTA-like spheroids, with or without microglia. In the absence of microglia, the incorporation of *SNCA* A53T dopaminergic neurons into VTA-like spheroids drastically changed the calcium oscillation pattern, as indicated by a significant reduction in peak rate and peak rise time, as well as increases in peak width, peak spacing, peak amplitude, and peak decay time (Fig. 6b–d and Supplementary Fig. 11b). However, the incorporation of microglia had only minor effects in WT VTA-like spheroids but significantly altered peak phenotypes in A53T VTA-like spheroids, making them more similar to WT VTA-like (MGL+) spheroids (Fig. 6c–e and Supplementary Figs. 11, 12). Specifically, when compared to WT VTA (MGL−), WT VTA (MGL+) had 22% decreased peak width (*p* < 0.0001), 14% decreased peak decay time (*p* = 0.42), and no significant differences in peak rate, amplitude, spacing, and rise time; while A53T VTA (MGL+) exhibited a 30% increase in peak rate (*p* < 0.0001), 40% decrease in peak width (*p* < 0.0001), 10% decrease in peak amplitude (ns, *p* = 0.25), 22% decrease in peak spacing (*p* < 0.0001), 6% increase in peak rise time (ns, *p* = 0.39), 20% decrease in peak decay time (*p* < 0.0001), compared to A53T VTA (MGL−). Importantly, while both WT and A53T VTA-like spheroids with microglia exhibit smaller peak width and peak decay time compared to those without microglia, the level of reduction is greater in A53T VTA spheroids, and as a result, there was no significant difference in 4/6 key peak parameters between microglia-containing WT VTA (MGL+) and A53T VTA (MGL+) (Fig. 6e and Supplementary Fig. 11b). These data suggest that the inclusion of functional, healthy microglia can reverse calcium activity profile changes observed in PD VTA-like spheroids with A53T dopaminergic neurons. To further define the cell-type specificity of this effect, we compared dopaminergic neuron-only spheroids with spheroids cultured with astrocytes, microglia, or both (Supplementary Fig. 11c). Astrocytes altered neuronal calcium activity but did not rescue the A53T-associated phenotype. In contrast, inclusion of microglia in either neuron–microglia co-cultures or neuron–microglia–astrocyte tri-cultures attenuated genotype-dependent differences in calcium oscillations, indicating that microglia were sufficient to normalize network activity under these conditions.

Next, we sought to investigate whether similar PD-associated phenotypes seen in A53T VTA-like spheroids can be replicated using an alternative PD disease-induction approach. We transduced WT VTA-like spheroids, with or without microglia, with either an adeno-associated virus (AAV) overexpressing A53T-α-synuclein or with an AAV-null empty vector on day 5 in culture, followed by regular maintenance until day 21 (Fig. 6f). Utilizing the MJFR-14-6-4-2 conformation specific antibody previously shown to detect α-synuclein aggregates^51^, immunohistochemistry revealed increased levels of total α-synuclein and α-synuclein aggregates in WT VTA-like spheroids, indicating efficient AAV-mediated α-synuclein expression by day 21 (Fig. 6g). When comparing the calcium oscillations in WT VTA-like spheroid transduced with AAV empty vector, there was no difference in peak rate but statistically significant reduction in peak width when microglia were present (Fig. 6h–j and Supplementary Fig. 12a). Importantly, we observed that overexpression of A53T-α-synuclein induces a reduction of peak rate and increase in peak width when microglia were not present. When healthy microglia were included in VTA-like spheroids overexpressing A53T-α-synuclein, there was an amelioration of the effects by A53T-α-synuclein overexpression, including significant increases in peak rate and reduction in peak width, so that there were no statistically significant differences in peak rate and peak width between VTA-like with microglia transduced with AVV empty vector and A53T-α-synuclein (Fig. 6h–j). To rule out a general effect of an overexpressed protein, we also transduced a separate set of WT VTA-like spheroids with AAV overexpression of tdTomato, a red fluorescent protein with no known neurological functions (AAV-tdTomato) at a matching MOI. We found no significant changes in either peak rate or peak width as a result of tdTomato overexpression in MGL− or MGL+ spheroids (Supplementary Fig. 12b, c), further indicating that the changes in calcium oscillation observed by overexpression of AAV-A53T-α-synuclein are likely due to disease-related pathology instead of a general protein overexpression, and that the observed microglial reversal of such phenotype related to effects produced by A53T-α-synuclein on network oscillations. We also examined baseline cytokine production in WT and A53T VTA-like spheroids in the absence or presence of microglia. Most cytokines in the panel were below the level of detection at baseline, with the exception of CCL2, consistent with Fig. 5. In the absence of microglia, A53T cultures showed a modest but non-significant increase in CCL2 relative to WT. Addition of microglia significantly reduced CCL2 levels in both genotypes, although CCL2 remained slightly elevated in A53T cultures compared with WT (Supplementary Fig. 12d). Together, these results suggest that the incorporation of healthy microglia can rescue A53T α-synuclein-associated functional deficits in VTA-like spheroids which are reflected in network activity alterations, further adding evidence to the value of NeuroMIMICS to investigate genetic and cellular drivers in neurological diseases, and as an assay platform for drug testing in the future.

## Discussion

In this study, we developed NeuroMIMICS, an HTS-compatible 3D tri-culture neural spheroid system that enables disease modeling and therapeutics development targeting both neural and microglial functionalities. The NeuroMIMICS system, which is assembled using human iPSC-derived microglia, astrocytes, and neurons with desired cell types and ratios, displays synchronized neural network activity and immune responses. We validated the functions of microglia in the NeuroMIMICS, including clearing of apoptotic cells, phagocytic activity, directed motility, and inflammatory response to internal and external stimuli. We also showed that the addition of microglia corrects disease-induced network activity phenotypes, including reversal of changes in spontaneous calcium oscillations induced by incorporation of PD-associated A53T dopaminergic neurons or expression of A53T alpha-synuclein via AAV.

The incorporation of either primary or iPSC-derived microglia into 3D neuronal cultures, including microglia-containing human brain organoids (MC-HBOs), has been previously reported (reviewed in Birtele et al.^52^). While 2D co-culture systems provide experimental accessibility, they lack the three-dimensional architecture necessary to recapitulate key aspects of cell–cell interactions in vivo. MC-HBOs offer increased physiological relevance; however, they still face several challenges that limit their utility for scalable and reproducible applications. Although some modified cerebral organoid generation protocols have been reported to allow the spontaneous generation of microglia in organoids^53^, most MC-HBO platforms rely on the generation of organoids followed by the subsequent addition of microglia via co-culture^24,54^. This approach typically requires large numbers of microglia per organoid (on the order of 1 × 10^5^–5 × 10^5^ cells) and can introduce more variability in microglial incorporation^12,18,20,21,55^. In contrast, the NeuroMIMICS platform incorporates microglia at the time of spheroid formation using substantially fewer cells (2 × 10^3^ per spheroid), enabling tighter control over microglia incorporation and reducing heterogeneity across spheroids (Supplementary Table 2). In addition, the large size of organoids frequently leads to hypoxic or necrotic core formation, which can confound the interpretation of microglial phenotypes through non-physiological activation states. NeuroMIMICS spheroids are maintained below 400 µm in diameter, minimizing diffusion limitations and reducing hypoxia-associated artifacts (Fig. 2d). Finally, while organoids provide structural complexity, they are generally limited by low synchronization of network activity and reliance on low-throughput, well-by-well functional assays. In contrast, NeuroMIMICS exhibit highly synchronized calcium dynamics that enable scalable, plate-based (384-well) quantitative functional readouts suitable for screening applications (Fig. 3).

Another advantage of this protocol is its versatility, allowing easy modification of the cell type composition and ratios in the seeding mixture to meet various experimental needs, which may better mimic the regional heterogeneity and morphophysiological variability of microglia in different brain regions^3^. The neuron:astrocyte ratio (10:1) was adopted from our previously published work^26^, in which this composition supported robust neuronal network formation and astrocyte-mediated modulation of activity. For the generation of NeuroMIMICS, the neuron: astrocyte:microglia seeding ratio (10:1:2) was selected based on functional validation, reported biological ranges, and experimental tractability. While defining a physiologically precise neuron:microglia ratio for the human VTA remains challenging, given the well-documented variability in microglial density across brain regions, developmental stages, and physiological states^56,57^, the ratio used here is within reported biological ranges. Postmortem human midbrain studies report microglial proportions ranging from approximately 3.8% in younger individuals to 9.7% in older adults^57^, and VTA IBA1^+^/TH^+^ ratios of 0.2–0.5 have been observed across the lifespan in aged C57BL/6J mice^58^. At the chosen ratio, microglia were present at sufficient levels to exert measurable effects on neuronal network activity, cytokine production, and neuroprotection. Importantly, NeuroMIMICS supports a range of seeding ratios (10:1:0–10:1:4; 10:2:0–10:2:4), demonstrating that cellular composition can be readily tuned to suit specific experimental contexts (Supplementary Fig. 2b).

Microglial densities, morphology, and transcriptomic signatures have been previously found to differ between brain compartments and between human vs. rodent brains^2,7–10,59^. Although the exact mechanisms mediating regional heterogeneity remain unclear, the resident microenvironments surrounding microglia likely play a critical role in establishing regional heterogeneity, as it has been reported that in vitro cultured microglia can regain in vivo genetic signatures and morphology after engraftment into the CNS of microglia-deficient mice^14,17,60^. These studies suggest that when developing 3D models with microglia for a certain disease state, relevant surrounding cell type composition is important. In light of these studies, combined with the knowledge that microglia express receptors for neurotransmitters including GABA^61^, glutamate^62^, and dopamine^63^, we therefore co-cultured microglia with different neuronal cell types to recreate activity-dependent plasticity in order to mimic some level of regional heterogeneity. By using pre-differentiated cells and a media composition suitable for the neural cell types of the cerebrum to be viable and functional (Supplementary Fig. 7), this proof-of-concept study demonstrates the feasibility of creating a physiologically relevant neural tissue model that includes microglia in an HTS-compatible plate platform, which could consider regional heterogeneity during drug discovery. Future studies will focus on characterizing microglial activation state and functionality differences between brain-region-specific NeuroMIMICS and delineating the underlying mechanisms to better understand how regional heterogeneity may play a role in neurological disease and therapeutics development.

During the formation of the NeuroMIMICS, embedded microglia displayed dynamic morphology changes over time indicating a change in activation state and tissue homeostasis, with the first week of culture characterized by an activated amoeboid shape followed by transition into a ramified shape with smaller cell bodies and branched with multiple primary and secondary processes, suggesting that the tri-culture spheroids were likely in a state of homeostasis around 3 weeks in culture as the resident microglia adopted a surveillance-associated morphology^64^. This observation was of importance given that microglia morphology is highly linked with their physiological roles; therefore, ramified microglia in day 21 spheroids signal an optimal homeostatic model with minimal endogenous influences, such as a necrotic core that could cloud the outcomes of assays involving exogenous stimuli. On the other hand, as microglia in this tri-culture platform are proven to be morphologically dynamic, evaluation of their morphology in states of disease or following drug treatment could facilitate our understanding of their precise roles. While this initial study focuses on mimicking physiological density as well as providing robust readouts, future studies may modulate microglia density to allow automated segmentation and characterization of microglial morphology and plasticity in healthy and disease-like NeuroMIMICS.

Drug discovery targeting microglial phagocytosis has been proposed as a potential therapeutic strategy for several neurodegenerative diseases^5,65^. In our model, tri-culture spheroids displayed higher phagocytic capacity compared to those without astrocytes, suggesting astrocytes play a role in microglia function (Fig. 2c and Supplementary Fig. 5a). We hypothesize that two factors might contribute to astrocyte enhancement of microglia function, including (1) the promotion of phagocytosis through intercellular crosstalk and (2) the facilitation of microglial aggregation within spheroids, resulting in higher microglial numbers. In agreement with these hypotheses, astrocytes have previously been implicated in the modulation of microglial phagocytosis during development through crosstalk between astrocytes and microglia^66^. While further studies are needed to clarify these findings, the critical role of astrocytes in innate and adaptive immunity, metabolic support to neurons, and their implication in learning and memory warrant astrocyte inclusion in a physiologically relevant model for disease modeling and therapeutic development.

Studies have shown that TLRs play a critical role in microglia-mediated inflammatory responses against not only exogenous pathogens but also neurodegenerative disease-associated aggregates^5,67,68^. Human primary cultures of microglia derived from postmortem human brain express detectable levels of *TLR1, TLR2, TLR3, TLR4, TLR5, TLR6, TLR7,* and *TLR8* and low levels or undetectable *TLR9* mRNA^49^. Transcriptomics analysis of the NeuroMIMICS showed the same TLR expression patterns (Fig. 4b). In this study, we found that poly I:C, PGN, or LPS treatments triggered the release of several cytokines in both VTA-like spheroids with microglia and microglia-only samples, but not in VTA-like spheroids without microglia, indicating the response is dependent on microglia presence (Fig. 5). Interestingly, poly I:C- or PGN-mediated cytokine response are different and significantly higher in VTA (MGL+) spheroids compared to MGL alone, suggesting that interaction with other cell types enhances microglia reactivity. While LPS induced a limited cytokine response, possibly due to the number of microglia present, previous studies consistently observed cytokine and chemokine release in microglia-containing organoids following LPS treatment^20,21,69,70^. It is possible that increasing the microglia count in future models may make LPS-induced responses detectable. Nonetheless, given their robust cytokine and chemokine release upon poly I:C stimulus, this NeuroMIMICS model could be suitable for viral infection modeling as well as antiviral screening.

In the CNS, glial cells, such as astrocytes and microglia, participate in network calcium oscillation by propagating calcium waves^71^ and modulating neuronal calcium activities via various mechanisms, such as responding to changes in neural synaptic activities^72^ and shaping neuroplasticity^73^. Correspondingly, we observed that the addition of either astrocyte or microglia appears to shift calcium oscillation patterns of single neuronal cell type spheroids, compared to neuron-only spheroids, whereas tri-culture spheroids exhibited calcium oscillation phenotypes similar to their respective neuron-astrocyte and neuron-microglia co-culture spheroids. This pattern supports the involvement of neuron-glia crosstalk during the formation of functional neural networks in spheroids, as well as possible overlapping roles of microglia and astrocytes in neural modulation. While further studies are needed to delineate the underlying mechanism of calcium oscillation shifts in the NeuroMIMICS system, the existence of such complex neural connections is critical in creating functional brain models for therapeutic development. Notably, microglia altered calcium oscillations in VTA-like but not PFC-like spheroids, pointing to possible regional differences, which have been mostly overlooked in existing drug screening models. It is worth noting that this study employed bulk RNA sequencing (RNAseq) to examine transcriptomic changes across the tissue resulting from the incorporation of microglia into the neural spheroid platform. While this approach successfully highlighted broad, tissue-wide transcriptomic changes, including neuroimmune functionality consistent with microglia-specific roles, a major limitation of bulk transcriptomics is the absence of single-cell resolution, which can mask stochastic gene expression and obscure cell-type-specific signals^74^. Consequently, while we were able to characterize global transcriptional shifts within the tissue that suggest the presence of microglia may promote a more refined neural network, we could not attribute specific changes to microglia versus neurons. Future studies will focus on optimizing tissue dissociation protocols to obtain representative, viable cells from these complex tissues without compromising the integrity or transcriptional profiles of sensitive cell populations, thus enabling robust scRNAseq analysis.

Given the importance of long-term network stability for modeling progressive neurodegenerative diseases, we next evaluated whether the NeuroMIMICS platform could be maintained beyond the standard 3-week culture period. We extended the culture period to 8 weeks and observed that NeuroMIMICS remained healthy and functionally active, exhibiting robust calcium oscillations throughout (Supplementary Fig. 13b, c). In contrast, microglia were detectable by IBA1 immunostaining only up to at least 6 weeks, with no detectable signal at 8 weeks (Supplementary Fig. 13a). Despite the persistence of neural network activity, increased variability over time suggested a decline in network stability (Supplementary Fig. 13d). Interestingly, monoculture glutamatergic-only spheroids completely lost spontaneous calcium oscillations by 8 weeks, whereas those co-cultured with astrocytes maintained activity, suggesting a neuromodulatory or neuroprotective role of astrocytes in the neural spheroids (Supplementary Fig. 13c). The current model supports robust tricultures up to at least 6-weeks, which is in line with other models reporting microglia loss over extended cultures^54^, although optimizing conditions to extend microglia persistence beyond this timeframe would broaden the platform’s applicability for modeling progressive diseases. To this end, a recent long-term adherent brain organoid model was shown to support microglia function up to 120 days post incorporation^75^.

We also demonstrated the neuroprotective role of healthy microglia in PD models, which affect functional activities that are used as HTS readouts. Specifically, two PD VTA-like (±MGL) spheroid models were developed by either replacing WT dopaminergic neurons with CRISPR-edited *SNCA* A53T dopaminergic neurons or by transducing WT VTA-like spheroids with AAV overexpressing A53T-alpha-synuclein. In spheroids without microglia, both PD disease models resulted in similar alterations in the spontaneous calcium oscillations compared to healthy controls, whereas the inclusion of healthy microglia prevented the PD-induced alterations. In A53T VTA cultures without microglia, CCL2 levels were slightly higher than in WT cultures, though not significantly (Supplementary Fig. 12d). When microglia were added, CCL2 levels dropped significantly in both genotypes, suggesting that microglia help suppress baseline inflammatory chemokine signaling and maintain immune balance, consistent with the previously reported role of microglia in maintaining immune quiescence. Since CCL2 has been linked to neuronal excitability and dopaminergic signaling^76–79^, this reduction may indirectly influence neural activity in the spheroids. However, A53T cultures with microglia still showed slightly higher CCL2 than WT, consistent with elevated CCL2 seen in PD patients^80^, implying that microglia may partially buffer genotype-related inflammatory effects but do not fully suppress them. Importantly, although the parallel normalization of calcium dynamics and reduction in CCL2 levels raises the possibility that microglia rescue neural activity via chemokine signaling, our data do not establish a causal relationship between these processes. CCL2 represents only one component of a complex neuroimmune signaling network, and microglia are known to influence neuronal activity through multiple mechanisms, including synaptic pruning, neurotransmitter regulation, and trophic support. Therefore, the observed association between reduced CCL2 and improved network function should be interpreted cautiously as a potential, but not definitive, mechanistic link. No significant differences in microglia morphology were observed between WT and A53T spheroids, which is in line with the similar neural activity phenotypes between WT and A53T VTA-like spheroids (Fig. 6e and Supplementary Fig. 12e). While these approaches enabled targeted modeling of PD-associated pathology in VTA-like circuits, future studies investigating AAV-mediated overexpression of *SNCA* A53T in additional brain-region-specific spheroids will help assess the generalizability of these findings. Another limitation of the present study is the focus on a single α-synuclein mutation model. While the A53T mutation represents a well-established genetic cause of PD, alternative mechanisms, such as *SNCA* duplication or triplication, result in increased wild-type α-synuclein expression and may produce distinct cellular phenotypes. Notably, A53T α-synuclein has been shown to induce a stronger pro-inflammatory microglial response compared to wild-type α-synuclein in primary microglial cultures^81^. Future studies incorporating these models will be important to distinguish mutation-specific effects from those driven by α-synuclein dosage and to determine whether microglial modulation of neuronal network activity generalizes across different genetic forms of the disease. In addition, an important open question is whether microglia carrying PD–associated mutations retain the capacity to modulate neuronal network activity in a similar manner. Patient-derived iPSC microglia have been shown to exhibit altered immune responses and functional properties, suggesting a potentially primed or dysregulated state^82–84^. It is therefore possible that disease-associated microglia may differ in their ability to support or restore neuronal function. Future studies incorporating microglia derived from A53T or *SNCA* triplication backgrounds could help determine how microglial genotype influences neuron–glia interactions and network stability, and clarify whether the observed rescue in the present study reflects a general property of microglia or is contingent on their genetic and functional state. Although additional studies are needed to more fully characterize healthy, PD, and microglial PD-corrected states, the neuroprotective role of microglia displayed in this model highlights the importance of incorporating the neural immune component into the drug discovery pipeline. This improvement of physiological relevance to the human brain may therefore improve translation from bench to clinic during drug discovery campaigns.

Although the NeuroMIMICS platform represents a significant advance over existing in vitro models, several limitations should be acknowledged. First, the system is intentionally modular to allow for the incorporation of diverse neural cell types; however, this flexibility also makes the model dependent on cell availability. In the present study, we relied on commercially available cell sources, which, while allowing immediate access by any lab regardless of iPSC expertise or in-house differentiation capabilities, constrained donor selection and resulted in mixed-donor spheroids, as well as limited the availability of disease-associated mutations to select neural subtypes. To begin assessing the impact of donor variability within this framework, we compared spheroids generated using distinct GABAergic neuron donors and did not observe significant differences in the measured outcomes (Supplementary Fig. 14), suggesting that donor-to-donor variability in this cell population does not strongly influence the phenotypes assessed here. However, this analysis was limited to a single cell type and set of conditions, and broader evaluation across additional cell types and functional readouts will be required to fully determine the impact of donor heterogeneity. Specifically, in the disease rescue experiments, only dopaminergic neurons with the *SNCA* A53T mutation were used, which was primarily driven by commercial availability of disease lines rather than experimental preference. While dopaminergic neurons represent the predominant cell type within the VTA-like spheroid and are the most directly implicated in PD pathology, restricting the mutation to a single population may limit the ability to fully assess cell-type-specific and non-cell-autonomous contributions to disease phenotypes. Ideally, future iterations of this model will employ isogenic donor lines across all neural cell types, including astrocytes and microglia, as this approach would enable a more comprehensive evaluation of intercellular interactions in the context of disease. These limitations may be addressed in future studies through either expanded commercial availability of disease lines or through the implementation of in-house differentiation protocols to generate fully isogenic, mutation-matched neural cell populations.

Finally, it is equally important for future studies to define the context of use of the NeuroMIMICS platform. While existing human brain organoid models continue to shed light on human brain development during healthy and disease conditions, this study describes the generation of an immunocompetent, reproducible tri-culture spheroid system that can be adapted to represent multiple brain regions and disease states, enables HTS for therapeutic discovery, development and neurotoxicity screening of new treatments for neurological diseases. Follow-up studies will focus on disease modeling, such as including hiPSC-derived microglia with disease-associated alleles and disease- and neuroinflammation-associated insults, to expand the application of the NeuroMIMICS system and their potential in therapeutics development.

## Methods

### Cells and donor information

Cryopreserved human iPSC-derived iCell neurons, iCell astrocytes, and iCell microglia were purchased from FUJIFILM Cellular Dynamics International (FCDI). All cells used in this study were provided by the manufacturer as fully differentiated and highly pure populations of cells that were quality checked for purity and cell profile via flow cytometry and RNAseq prior to shipping.

Cell lines used in this study include two donors across cell lines used, based on commercial availability: Donor ID# 01434, Caucasian female (age <18) with a healthy phenotype and Donor ID# 01279, Caucasian male (aged 50–59) with a healthy phenotype. Wildtype cell lines used includes: iCell astrocytes (Cat# R1092, Donor ID# 01434, differentiated from fibroblast), iCell GlutaNeurons (Cat# C1033, Donor ID# 01279, differentiated from blood-derived iPSCs, only use if gluta purity is >80%), iCell GABANeurons (Cat# C1012, Donor ID# 01434, differentiated from fibroblast), iCell DopaNeurons (Cat# C1028, Donor ID# 01279, differentiated from blood-derived iPSCs), and iCell microglia (Cat# C1110, Donor ID# 01279, differentiated from blood-derived iPSCs using a protocol previously developed by Blurton-Jones laboratory^12^). Two vials of GFP-labeled iCell microglia (no-cat, Donor ID# 01279) were purchased from FCDI for the live imaging experiments used for initial assay optimization only; data acquired from GFP-microglia were not used in quantification or statistical analysis. Two vials of iCell GABANeurons (Cat# C1008, Donor ID# 01279) were used to compare donor differences.

To investigate PD-related phenotype, iCell DopaNeurons PD *SNCA* A53T HZ (Cat# C1113, Donor ID# 01279), which encodes a heterozygous A53T allelic variant in the gene for *SNCA* and isogenic to iCell DopaNeurons, was used.

### Cell thawing

Each cell line used in the study was thawed according to the manufacturer’s recommendation.

For iCell Microglia, each cryovial was placed in a 37 °C water bath for exactly 3 min before the content was transferred into a 15 mL conical tube pre-filled with 3 mL microglia thawing media containing iCell glial base medium (FCDI, Cat # M1034) and 1× iCell neural supplement C (FCDI, Cat # M1046). Each cryovial was rinsed with an additional 1 mL of microglia thawing media, which was added to the cell suspension before gently mixing. Conical tubes containing iCell microglia were centrifuged at 1000 × *g* for 10 min. The supernatant was aspirated, and each cell pellet was resuspended in 5 mL microglia monoculture media containing iCell glial base medium, 1× iCell microglia supplement A (FCDI, Cat # M1036), 1× iCell microglia supplement B (FCDI, Cat #M1037), and 1× iCell neural supplement C. Each tube of resuspended microglia (>1.5 M cells/vial) was seeded into 2 wells of a Costar 6-well Clear Flat Bottom ULA plate (Corning, Cat # 3471) with approximately 2.5 mL cell resuspension/well and cultured for 3 days in the incubator. Three days later, microglia in each well were collected using the waterfall technique. Briefly, microglia monoculture media was aspirated and re-dispense into each well of the plate tilted at a 45-degree angle 4–5 times to wash the adhering cells off the plate. The microglia resuspension was then transferred into a new 50 mL conical tube. Two milliliters pre-chilled DPBS (no calcium no magnesium, Gibco, Cat # 14190144) was added back to each well, and the plate was placed in 4 °C for 10 min before bringing back to the BSC and repeat the waterfall technique for cell collection. The process was repeated 3–4 times until most microglia have been collected into the conical tube. Conical tubes containing iCell microglia were centrifuged at 1000 × *g* for 10 min. The supernatant was aspirated, and the cell pellet was resuspended in “tri-culture seeding media” containing iCell base medium 1 supplemented with 1× neural supplement A and 1× iCell microglia supplement A (FCDI, Cat # M1036), until further use.

For iCell GABANeurons and iCell Astrocytes, cryovials were placed in a 37 °C water bath for exactly 3 min before the contents of each vial were transferred into separate conical tubes. For iCell GlutaNeurons and iCell DopaNeurons, cryovials were thawed in a 37 °C water bath for exactly 2 min. Different thawing media were used in the following steps: (a) thawing media for iCell GABANeurons and iCell astrocytes: iCell base medium 1 (FCDI, Cat # M1010) supplemented with 1× neural supplement A (FCDI, Cat # M1032); (b) thawing media for iCell GlutaNeurons: BrainPhys neuronal medium without phenol red (STEMCELL Technologies, Cat # 05791) supplemented with 1× neural supplement B (FCDI, Cat # M1029), 1× nervous system supplement (FCDI, Cat # M1031), 1% N2 supplement (Thermo, Cat # 17502048), and 0.1% laminin (Invitrogen, Cat # 23017-015); (c) thawing media for iCell DopaNeurons: iCell base medium supplemented with 1× neural supplement B and 1× nervous system supplement. One milliliter of thawing media corresponding to each cell type was used to rinse each cryovial, then dispense to the conical tubes with cell suspension in a drop-wise fashion. Additional 8 mL of thawing media was slowly added to each tube before gently mixing the cell suspension. iCell GABANeurons and iCell Astrocytes were then centrifuged at 300 × *g* for 5 min while iCell GlutaNeurons and iCell DopaNeurons were centrifuged at 400 × *g* for 5 min. The supernatant was discarded, and cell pellets were resuspended in the tri-culture seeding media. Resuspended cells were counted using a Countess Cell Counter (ThermoFisherScientific) before seeding.

### Media optimization for tri-culture neurons, astrocytes, and microglia

Two independent 2D cultures were done for the optimization of the BrainPhys-based Tri-culture media. Microglia were thawed on day −3, immediately seeded in a 6-well ULA flat-bottom plate and allowed to recover for 3 days in the incubator. On day 0, microglia were collected using the waterfall technique described above, while glutamatergic neurons and astrocytes were freshly thawed. All cells were resuspended in tri-culture seeding media and seeded in a 96-well flat-bottom plate pre-coated with 0.01% poly-L-ornithine hydrochloride (PLO, Sigma, P2533) in PBS for 1 h at room temperature, followed by overnight coating in 3.3 µg/ml Laminin (Invitrogen, 23017-015) solution in PBS at 4 °C. For media optimization experiments, glutamatergic neurons, astrocytes, and microglia were seeded at 40k: 8k: 8k per well with 100 µL tri-culture seeding media (iCell base medium 1 supplemented with 1× neural supplement A and 1× iCell microglia supplement A) and placed in the incubator for 24 h to allow cells to settle on the bottom of the wells. On day 1, 100 µL of BrainPhys-based testing media (BrainPhys without phenol red supplemented with 1× N2, 1× B27, 20 ng/mL BDNF, 20 ng/mL GDNF, 1 µg/mL laminin) with different supplement combinations (1 mM cAMP, 200 µM ascorbic acid, 2 ng/mL TGF-β2, 100 ng/mL M-CSF, 1.5 µg/mL cholesterol) was added to assigned wells. Starting day 3, half media changes with testing media were done every other day until day 21. On day 21, cell cultures were fixed with 4% paraformaldehyde (PFA) for 15 min at room temperature, then washed with PBS before proceeding to immunofluorescence staining.

### Generation of microglia-containing neural spheroids

On day −3, cryopreserved iCell microglia were thawed and seeded in 6-well Clear Flat Bottom ULA plates (Corning, Cat # 3471) as described above to allow microglia to recover and stabilize from the thawing process.

On day 0, 384-well ultra-low attachment (ULA) round-bottom plates (Corning, Cat # 3830) were pre-treated with 40 µL/well anti-adherence rinsing solution (STEMCELL Technologies, Cat # 07010) and centrifuged at 1000 × *g* for 5 min. Anti-adherence rinsing solution was then aspirated, and 80 µL warm BrainPhys without phenol red was used to wash each well. The media was again aspirated, and 40 µL warm BrainPhys without phenol red was added to each well. The pre-treated plates were left at room temperature inside the BSC until further use. After the pre-treatment of the plates, neurons and astrocytes were thawed, and microglia were harvested as previously described. Each cell type was resuspended in tri-culture seeding media (iCell base medium 1 supplemented with 1× neural supplement A and 1× iCell microglia supplement A) to achieve a cell suspension containing 5e5 viable cells/mL. In all experiments, cell seeding was normalized based on the number of viable cells per cell type, and cell lots with similar viability ranges were selected. Cell types required in each spheroid type were then mixed in fresh 50 mL conical tubes, and tri-culture seeding media was added to achieve the final concentration needed for each spheroid type. Other than spheroids used for cytokine collection, neural spheroids in this study were seeded according to the cell type composition listed in Table 1, with a ratio of 10:1:2 for total neurons:astrocytes:microglia. Fifty microliters of mixed cell suspension was manually dispensed into anti-adherence pre-treated 384-well round-bottom ULA microplates using a 16-channel multichannel Finnpipette (Thermo Fisher Scientific). Plates were then sealed with parafilm and centrifuged at 350 × *g* for 10 min to pull cells to the bottom of the plate. On day 1, 40 µL neural spheroid tri-culture maintenance media (BrainPhys without phenol red supplemented with 1× N2, 1× B27, 20 ng/mL BDNF, 20 ng/mL GDNF, 1 µg/mL laminin, 200 µM ascorbic acid, 2 ng/mL TGF-β2, 100 ng/mL M-CSF, and 1.5 µg/mL cholesterol) was added to achieve 90 µL total media in each well. Starting on day 3, half-media changes using the neural spheroid tri-culture maintenance media were done every other day prior to testing.

### AAV transduction

To overexpress A53T alpha-synuclein in spheroids, AAV1/2-CMV/CBA-human-A53T-alpha synuclein-WPRE-BGH-polyA and the control AAV, AAV1/2-CMV/CBA-null/empty-WPRE-BGH-polyA (Charles River, Cat # GD1001-RV and GD1004-RV) were added to spheroid-containing wells 5 days after spheroid generation at MOI = 5e5 via half media change. To rule out a general effect of an overexpressed protein, AAV2-CAG-tdTomato (Addgene, Cat # 59462-AAV2) was transduced at matching MOI.

### Immunofluorescence staining

2D cell cultures were fixed for 15 min with 4% PFA in PBS and washed 3 times with PBS. Immunofluorescence staining of 2D cultures was done via manual pipetting, and all incubation steps occurred on a shaker. Permeabilization was done by using 0.5% Triton X-100 (Sigma, cat # X100) in PBS for 15 min at room temperature. The cells were washed for 3 times with PBS and blocked with 1× blocking solution consisting of 5% normal goat serum (NGS; Millipore Sigma, Cat # S26-LITER) and 2% bovine serum albumin (BSA; Fisher, Cat # BP1605) in PBS for 1 h in room temperature. Cells were then incubated with desired primary antibodies diluted in 1× blocking solution overnight at 4 °C. The antibodies used are listed in Supplementary Table 3. The next day, cells were washed 3 times with PBS and then incubated with desired secondary antibodies at 1:1000 diluted in 1× blocking solution for 2 h at room temperature, blocked from light. The cells were then washed 3 times with PBS before counterstained with DAPI solution for 10 min at room temperature. After a final wash with PBS, the plates were sealed and either directly imaged or stored at 4 °C until imaging.

Spheroids were fixed with 4% PFA in PBS overnight in 384-well plates at 4 °C and washed with PBS the following day by doing “half changes,” where half of the PFA solution was removed and exchanged with PBS, for a total of four times. On the final wash, PBS with 0.1% sodium azide (Sigma, Cat # S2002) was added for spheroid preservation. Plates were sealed and stored at 4 °C until further use. During the immunofluorescence staining procedure for 3D neural spheroids, all liquid removal steps were performed via “half changes” as described above, and all incubation steps occurred on a temperature-controlled shaker with a reservoir filled with ddH_2_O placed inside to minimize evaporation in the microplates. First, PBS with 0.1% sodium azide was washed with PBS after 3 “half changes,” leaving 30 µL PBS per well. For blocking, equal volume (30 µL/well) of 2× blocking solution consisting of 10% normal goat serum, 4% bovine serum albumin, and 1% Triton X-100 in PBS was added to each well with 30 µL PBS to achieve a final concentration of 5% normal goat serum, 2% bovine serum albumin, and 0.5% Triton X-100 in 60 µL/well PBS. Spheroids were blocked in 1× blocking solution for 1 h at 37 °C or overnight at 4 °C. After blocking, 30 µL/well blocking solution was removed, and 30 µL/well primary antibodies (2×) in 1× blocking solution were added for the desired 1× final dilution. The antibodies and dilutions used are listed in Supplementary Table 3. Spheroids were incubated with primary antibodies overnight at 37 °C. Spheroids were then washed with 0.3% Triton X-100 in PBS three times, followed by an additional three washes with 15-min incubations. Thirty microliters/well secondary antibodies (2×) were made in 2× blocking solution and added via a “half change” to achieve a final dilution of 1:600. Spheroids were incubated with secondary antibodies at 37 °C overnight, followed with three washes of 0.3% Triton X-100 in PBS. For counterstaining, spheroids were incubated in DAPI solution for 30 min before washed with PBS.

Following immunostaining, tissue clearing was done by carefully aspirating all PBS from each spheroid-containing well and replacing with 60 µL/well ScaleS4 Tissue clearing solution. ScaleS4 contains 40% D-sorbitol (Sigma, Cat # S6021), 10% glycerol (Sigma, Cat # G2289), 4 M urea (Sigma, Cat # U5378), 0.2% Triton X-100, 15% DMSO (Sigma, Cat # D2650) in UltraPure water (Invitrogen, Cat # 10977-015)^85^. Spheroid plates with ScaleS4 clearing solution were sealed and placed on the shaker at 37 °C overnight, protected from light. The following day, the plates were stored at 4 °C until imaging.

### Phagocytosis assay for 3D spheroids

Spheroids cultured for 21 days were transferred to new 384-well ULA round-bottom plates with 30 µL media per well via manual pipetting on the day of the experiment. The pHrodo red *S. aureus* BioParticles conjugate for phagocytosis (ThermoFisherScientific, Cat. No. A10010, 2 mg) were first resuspended in 2 mL tri-culture media via vortex to achieve a 1 mg/mL stock solution. The stock solution was sonicated for 10 min before further diluted in warm tri-culture media as a 2× solution immediately before transferring 30 µL to each spheroid, achieving a final working concentration of 100 µg/mL. For mock control groups, 30 µL warm tri-culture media were added to each well. Media in each well was gently pipetted up and down once every hour to ensure even distribution of the bioparticle. The 384-well plates were placed back in the incubator, and a subset of spheroids from each group were fixed with 4% PFA at 2, 4, 6, and 8 h after pHrodo or mock treatment. The spheroids were incubated in 4% PFA overnight at 4 °C. The next day, spheroids were washed with PBS and proceed with downstream immunofluorescent staining and imaging.

### Dead cell labeling with propidium iodide (PI)

Propidium iodide (PI, Thermo Fisher Scientific, Cat # P3566) was used to label dead cells on live spheroids. Spheroids were transferred to new 384-well ULA round-bottom plates with 30 µL/well media via manual pipetting on the day of the experiment. PI solution and Hoechst 33342 (Thermo Fisher Scientific, Cat # 62249) were first diluted in warm tri-culture media as a 2× staining solution. Then 30 µL of the 2× staining solution was added to each well to achieve final concentrations of 1.5 μM PI and 10 μM Hoechst 33342. Spheroids were incubated at 37 °C for 30 min prior to live cell imaging using Opera Phenix plus high-content screening system (Revvity).

### Microglia depletion assay

Pexidartinib (PLX-3397, TOCRIS, Cat # 7590) was used for microglia depletion in tri-culture spheroids after 21 days in culture. PLX-3397 was reconstituted in DMSO as a 10 mM stock and further diluted in warm tri-culture media to 2× desired working concentration (to achieve final concentration at 0.01, 0.04, or 0.12 µM). On the day of the assay, half of the culture media was removed from each well and replaced with equal volume of 2× PLX-3397 containing media. Spheroids were treated with PLX-3397 (or DMSO as vehicle control) for a total of 96 h, with one-half media change with 1× PLX-3397 around 48 h post-treatment. Spheroids were fixed with 4% PFA after a 96-h of treatment.

### Microglia migration assembloid assay

To assess the mobility of microglia, two sets of spheroids were generated and cultured separately in 384w ULA round-bottom plate until day 21: “origin spheroids” were tri-culture spheroids generated with glutamatergic neurons, astrocytes, and microglia, while “destination spheroids” were co-culture spheroids of glutamatergic neurons and astrocytes. On day 5 after spheroid generation, “destination spheroids” were transduced with AAV2-CAG-tdTomato (Addgene, Cat# 59462) at MOI = 1e5 to allow the visual distinction between the two types of spheroids once the assembloid is formed. On day 21, each “starter spheroid” was first pre-treated with 10 µM PSB0739 (TOCRIS, Cat # 3983) or ddH_2_O as vehicle for 4 h before transferring to the same round-bottom well as a tdTomato-expressing “destination spheroid”. Plates were centrifuged at 300 × *g* for 1 min followed by a visual check to ensure both the “starter spheroid” and “destination spheroids” rest at the bottom of the well. After a 12-h incubation in the incubator, the assembloids were fixed with 4% PFA and proceeded for immunostaining and imaging.

### RNA isolation and RNA-sequencing

Spheroids were grown in 384-well round-bottom ultra-low attachment microplates as previously described for RNA extraction and submission to Genewiz from Azenta Life Sciences (New Jersey, USA) for whole RNA sequencing. Approximately 75 spheroids were used per sample; samples were collected in triplicate, and the extracted total RNA was submitted for RNA sequencing (*N* = 3 per condition from 1 experiment, Genewiz). For RNA extraction, spheroids were collected in 2 mL tubes and centrifuged for 5 min at 500 × *g,* and the supernatant was discarded. The remaining cell pellet was lysed using TRIzol™ Reagent (Cat # 15596026) according to product input sample requirements of 5 × 10^6^ cells per 1 mL of TRIzol™ Reagent. The samples were stored frozen at −80 °C as a stopping point prior to completion of RNA isolation. Total RNA was purified using Direct-zol™ RNA MiniPrep Kits according to the manufacturer’s instructions (Zymo Research, Cat # R2052). Concentration and quality of RNA was determined to match the guidelines of Genewiz from Azenta Life Sciences for RNA sequencing (ultra-low input), and concentration ≥20 ng/μL total RNA resuspended in Nuclease-free water. Sequencing was done with MiSeq, PolyA-selected and non-strand specific using paired-end 250 bp (2 × 250). The data output was 20 M read pairs (40 M raw reads).

Differential expression analysis was conducted in R using the DESeq2 package^86^. Genes with fewer than 10 total counts across all samples were excluded prior to analysis. DESeq2 normalized count data using its median-of-ratios method and modeled gene expression using a negative binomial distribution. Genes were considered differentially expressed if they had an adjusted *p*-value (false discovery rate, FDR) < 0.05 and an absolute log2 fold change > 1. Pathway enrichment analysis of DEGs was performed using the clusterProfiler package^87,88^, leveraging Gene Ontology (GO) annotations. Enrichment results were filtered at FDR < 0.05 and visualized using dot plots. Visualization for data analysis was generated using R packages: EnhancedVolcano (https://bioconductor.org/packages/devel/bioc/vignettes/EnhancedVolcano/inst/doc/EnhancedVolcano.html), ggvenn (https://github.com/yanjunjie/ggvenn).

Cell-type proportions in bulk RNA-seq samples were estimated using the MuSiC algorithm, with the HNOCA single-cell RNA-seq dataset as the reference ^35,36^. Differential expressions among VTA, VTA+MGL, and MGL samples were performed using DESeq2 (design: ~condition). Intrinsic MGL signature genes were defined as those significantly differentially expressed between MGL and VTA samples (Benjamini–Hochberg adjusted *P* < 0.05, |log2FC | ≥ 0.5)

### qRT PCR

Total RNA was extracted as described above. Quantitative RT-PCR was performed using the One Step PrimeScript III RT-PCR Kit (TaKaRa, Cat# RR600A) according to the manufacturer’s instructions. Reactions were prepared at a total volume of 10 µL, with each sample run in triplicate technical replicates.

Amplification was carried out on a Bio-Rad CFX384 Real-Time PCR System under standard cycling conditions recommended by the manufacturer. Fluorescence signals were collected during each cycle, and relative gene expression levels were calculated using the comparative Ct (ΔΔCt) method with normalization to GAPDH. Primers used are shown in Supplementary Table 4.

### Cytokine assay

VTA-like spheroids with approximately 15k neurons, 1.5k astrocytes, with or without 3k microglia per spheroid were generated using the protocol described above. Microglia-only samples were seeded with 3k microglia/well in the same 384-well ULA round-bottom plates. All samples were maintained for 20 days in 90 µL of tri-culture media. On day 20, 60 µl of the media was removed, and 30 µL of fresh media containing 2× concentration of the different TLR stimuli were added to 30 µl of the remaining media to generate a final 1× concentration. Final concentration of 10 µg/ml lipopolysaccharide (LPS, Millipore Sigma, Cat#L3024-5MG), 10 µg/mL polyinosinic-polycytidylic acid-high molecular weight (poly (I:C) HMW, InvivoGen, Cat # tlrl-pic), 10 µg/mL peptidoglycan from *S. aureus* (PGN, InvivoGen, Cat # tlrl-pgns2), or 5 µM of class C CpG oligonucleotide (CpG, InvivoGen, Cat # tlrl-2395) were added to the spheroids and incubated for 24 h before collecting the conditioned media. After 24 h, 50 µl from each well were collected, and the supernatant from 3 individual spheroid wells of each condition were pooled into 1 sample for each experiment. In each experiment, a total of *N* = 2 samples per condition were collected and analyzed. The conditioned media were collected and frozen at −80 °C until ready for the Luminex Multiplex Assay.

Luminex Multiplex assay kits were customized and purchased from Bio-Techne R&D Systems. Selected cytokine and chemokine concentrations in the spheroid culture supernatants were determined by Bio-Techne Human Premixed Multi-Analyte Luminex Kit and Luminex FLEXMAP 3D instrument following the manufacturer’s instructions. The data obtained were analyzed using Bio-Plex Manager software. The analytes and their corresponding sample dilution factors in this study includes: TNF (1:2), IL-6 (1:2), MMP-9 (1:2), MMP-3 (1:2), MMP-1 (1:2), IL-10 (1:2), CCL2 (1:2), MMP-8 (1:2), IL-1beta (1:2), IL-1alpha (1:2), IL-8 (1:5), and CXCL10 (1:5). Cytokine values below the assay’s limit of detection (<OOR) were labeled in Supplementary Data File and considered not detected and replaced with the value zero prior to statistical analysis.

### Cell-Titer-Glo 3D viability assay

CellTiter-Glo 3D Cell Viability Assay (Promega, Cat# G9682) was used according to the manufacturer’s instructions. CellTiter-Glo 3D Reagent was thawed overnight at 4 °C and brought to room temperature for at least 30-min before use. Half of the media in each well was aspirated and replaced with equal volume of CellTiter-Glo 3D Reagent. The spheroid plate was shaken vigorously for 5-min at room temperature, followed by a 25-min incubation period on the bench at RT. Luminescence was read using a PHERAstar FSX microplate reader (BMG LabTech) to measure the amount of adenosine 5′-triphosphate (ATP) present.

### Cal6-FLIPR assay and data processing

Spheroids were cultured for 21 days in 90 µL media per well prior to the Cal6-FLIPR assay. On the day of the Cal6-FLIPR assay, each bottle of Cal6 dye was equilibrated to room temperature before mixing with 10 mL room temperature tri-culture media. The mixture was vortexed for 2 min until the dye was completely dissolved. Sixty microliters media was carefully aspirated from each well without disturbing the spheroid and replaced with 30 µL Cal6 mix. The microplates were loosely wrapped in foil and placed back in the incubator for 2 h. After 2 h of incubation in Cal6 dye, plates were transferred into a fluorescent imaging plate reader (FLIPR) penta high-throughput cellular screening system (Molecular Devices) with the platform pre-warmed to 37 °C.

Spontaneous calcium oscillatory data was recorded through ScreenWorks 5.1 (Molecular Devices). Standard filter sets were used for Cal6 imaging with excitation set at 470–495 nm and emission at 515–575 nm. Exposure time was set to 0.03 s with 50% excitation intensity. Reading interval was set to 0.6 s, with 500 reads for a 5-min recording. Initial extraction of 18 peak parameters (mean peak amplitude, peak amplitude standard deviation (SD), peak count, mean peak rate, peak rate SD, peak spacing, peakspacing SD, mean number of early-depolarization like event (EAD-like) peaks per well, calcium transient duration (CTD) at 50% (peak width at 50% amplitude), CTD at 90% (peak width at 90% amplitude), rise slope, rise slope SD, mean peak rise time, peak rise time SD, decay slope, decay slope SD, mean peak decay time, and peak decay time SD) was done via the PeakPro 2.0 module within ScreenWorks (Molecular Devices) as previously described^89^. We specifically focused on 6 key parameters with coefficients of variation below 30% that can best describe the peak phenotypes, which included mean peak amplitude (PkA), mean peak rate (PkRate), peak spacing (PkSp), peak width at 50% (PkW50), peak rise time, and peak decay time (Supplementary Table 1).

All data were normalized to the average of control wells within each trial before combining them for statistical analysis. Each group represented on the radar plots shows the means of each parameter in comparison to controls, which were always averaged to 100%. Bar plots with individual values are reported as mean ± SEM.

### Image acquisition and analysis

Representative images of spheroids following immunofluorescent staining were captured using a Leica confocal microscope with a 10× air or a 25× water objective. Images acquired from the Leica Confocal were all processed using FIJI. For the phagocytosis experiment, a 25× z-stack containing 6 images with a z-step of 30 µm was captured to cover half of each spheroid with minimal repeat imaging of the same cell. Filter settings for Alexa Fluor™ 568 were used to image internalized pHrodo BioParticles conjugates. Batch analysis of each single plane image was done in FIJI. An intensity threshold was selected to reject the black background and use as a mask for the spheroid. The analyze particles function was used to identify pHrodo+ objects, and the sum of identified objects from the 6-plane z-stack was used for statistical analysis. For the assembloid experiment, a multi-channel 10× z-stack containing 37 images with a 5 µm z-step was captured for each assembloid. A multi-TIFF of the z-stack was created for each spheroid using FIJI for manual quantification of migrated microglia. The spheroid within the assembloid with tdTomato-expressing cells was identified as the “destination spheroid,” and the spheroid without tdTomato-expressing cells was identified as the “origin spheroid.” The number of IBA1-positive cells in “destination spheroids” were manually marked and counted by researchers blinded to the treatment.

To estimate the number of IBA1-positive cells in each spheroid, the ImageXpress Micro Confocal High-Content Imaging system (Molecular Devices) was used to automatically acquire a z-stack of approximately 250 µm deep into each spheroid (z-step = 2 µm) in all channels used in immunofluorescent staining with a 20× Water Apo objective. The MetaXpress analysis software (Molecular Devices) was used for the segmentation of nuclei and IBA1-positive cells in 3D. Specifically, an intensity threshold was selected based on the DAPI channel to generate a mask for the spheroid, therefore rejecting any debris or non-integrated microglial signal. The cell scoring module was used to identify microglia as cells that were labeled by both DAPI (nucleus) and IBA1 (cytoplasm). For quality control, spheroids with less than 3000 total number of DAPI-positive objects identified were eliminated from each group. The total numbers of microglia (DAPI+/IBA1+ objects) in the remaining spheroids were used for statistical analysis. To estimate the number of PI- labeled dead cells in each spheroid, the Opera Phenix Plus spinning disk confocal (Revvity) was used to automatically acquire a z-stack of each spheroid, and the Harmony high-content image analysis software (Revvity) was used for quantification.

For live cell imaging, 384-well microplates with spheroids incubated in PI and Hoechst 33342 were placed in the Phenix Plus with the stage pre-warmed to 37 °C with 5% carbon dioxide circulating. A filter setting with excitation of 561 nm and emission of 570–630 nm was used to image PI, and a filter setting with excitation of 375 nm with emission of 435–480 nm was used to image Hoechst 33342. Z-stacks of 91 images were collected with a 20× air objective (NA 0.4) using a 1 μm z-step. The Harmony analysis software was used for the masking of the spheroid based on Hoechst 33342 signal and the automated segmentation of nuclei and PI-positive objects in 3D analysis mode.

### Graphical plots

Biorender was used to create schematics of experimental procedures. GraphPad Prism 9.4.1 was used to make time series plots of calcium oscillations and column graphs. For radar plots showing multiparametric peak alterations across six peak parameters, Microsoft Excel was used based on the means of single neuron spheroids, MGL- spheroids, or wild-type mock spheroids as 100%.

### Statistics and reproducibility

Statistical analyses were performed using GraphPad Prism 9.4.1 and RStudio. Outliers were identified using Grubbs’ test (*α* = 0.05) and excluded from subsequent analyses. Data distributions were first assessed for normality, followed by evaluation of residual normality where applicable. Based on these assessments, appropriate parametric or non-parametric statistical tests were applied as indicated in the corresponding figure legends.

Data are presented as mean ± SEM for column plots and as mean values for radar plots. Statistical significance was defined as *P* < 0.05. All raw data used to generate the figures are provided in the Supplementary Data File. Identified outliers are indicated in blue and marked with an asterisk (*) in the dataset. Data used in the main figures were each collected from at least three independent trials except for bulk RNAseq (*n* = 3 from one trial) and cytokine analysis (two independent trials in which supernatants from 3 spheroids were pooled as 1 sample, *n* = 2 samples were collected from each trial). Specifically, data from Figs. 1F and 2C, E, H, were each collected from three independent experiments with *n* = 4–8 technical replicates per experiment; data from Fig. 3 were collected from three independent experiments with *n* = 16 technical replicates per experiment; data from Fig. 6 were each collected from four independent experiments with *n* = 8–16 technical replicates per experiment.

## Supplementary information

Supplementary Information

Description of Additional Supplementary Files

Supplementary Data

nr-reporting-summary

## Data Availability

RNAseq datasets generated during and/or analyzed during the current study are deposited to Gene Expression Omnibus, a public functional genomics data repository, under GEO Dataset GSE335200. All other data supporting the findings of this study are available within the paper and its Supplementary Information.
