# Supplementary material for: Microglia integrated neural spheroids enable neuroinflammatory responses and correct network dysfunction induced by alpha-synuclein mutation
Source: Commun Biol. 2026 Jul 15;9:1482. doi: 10.1038/s42003-026-10696-w (PMC13740307; doi:10.1038/s42003-026-10696-w)
Supplement: Supplementary file 1 [file 42003_2026_10696_MOESM1_ESM.pdf]

Supplementary Material

Microglia integrated neural spheroids enable neuroinflammatory responses and correct network dysfunction induced by alpha-synuclein mutation

Jiajing Zhang[1], Yi Wei Lim[1], Angelica Medina[1], Yu-Chi Chen[1], Yining Irene Wang[1], Martin Carrasco Carvajal[1], Marc Ferrer[1]*, & Emily M. Lee[1]*

[1]Division of Preclinical Innovation, National Center for Advancing Translational Sciences (NCATS), National Institutes of Health, 9800 Medical Center Drive, Rockville, MD, 20850, USA

*Author to whom any correspondence should be addressed.

E-mail: Marc.ferrer@nih.gov, Emily.lee@nih.gov

a

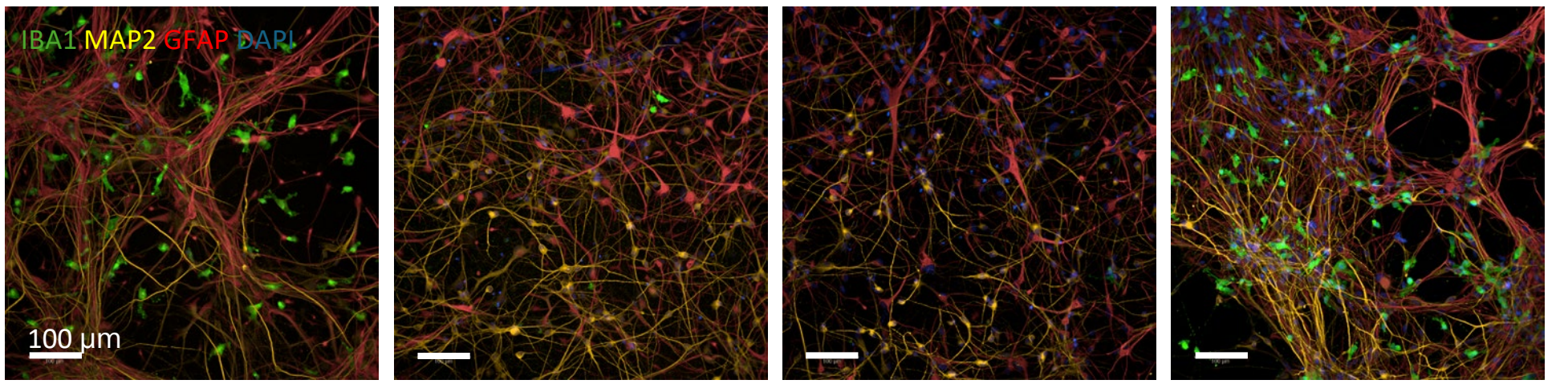

Media	A	B	C	D (tri-culture media)
Manufacturer supplied media *(not compatible for neural electrophysiology measurement)*	+			
BrainPhys media		+	+	+
1X N2, 1X B27, BDNF (20 ng/mL), GDNF (20 ng/mL), Laminin (1 µg/mL)		+	+	+
cAMP (1 mM)		+	+	
ascorbic acid (200 µM)		+	+	+
TGF-β2 (2 ng/mL)			+	+
M-CSF (100 ng/mL)			+	+
Cholesterol (1.5 µg/mL)			+	+

Supplementary Figure 1. Media optimization for tri-culturing human iPSC-derived neurons, astrocytes, and microglia. (**a**) Representative images (20X) of 21-day tri-cultures seeded with glutamatergic neurons, astrocytes, and microglia in flat bottom plates as 2D cultures and maintained in A, B, C, D, four different media. Immunostaining against microglia marker (IBA1), neuron marker (MAP2), astrocyte marker (GFAP), as well as DAPI counterstaining were performed to visualize the present of all three cell types in media tested. Scale bar = 100 µm.

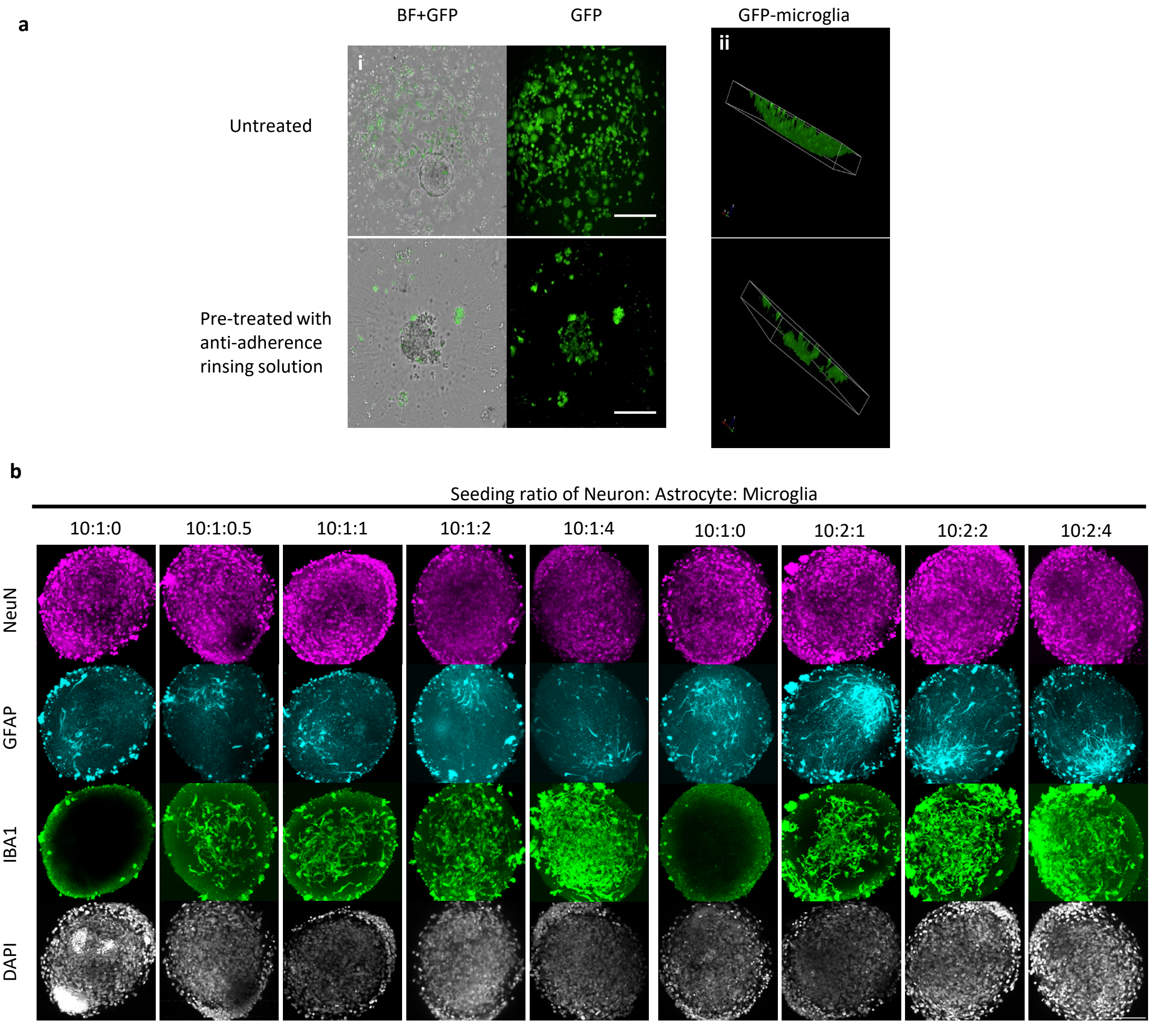

Supplementary Figure 2. Microglial aggregation into neural spheroids under reduced surface tension with anti-adherence rinsing solution and with different seeding ratios.

(**a**) Representative bright field (BF) or GFP-channel images of tri-culture spheroids 7 days after seeded in (top) untreated 384w ULA round bottom plates or (bottom) same microplate pre-treated with anti-adherence rinsing solution prior to seeding. (**a-i**) showing maximum projections of representative wells showing the localization of GFP-tagged microglia with reference to the cell aggregation shown in bright field images. (**a-ii**) 3D rendering from the same confocal z-stacks showing GFP-microglia attached to the bottom of the untreated well but aggregated in the pre-treated well. 10X objective was used. Scale bar = 500 µm.

(**b**) Representative images of individual spheroids seeded with varying ratio of Neuron : Astrocyte : Microglia. Antibodies used to identify cell-types include NeuN (neurons), GFAP (astrocytes), and IBA1 (microglia), with DAPI used as a marker for nuclei. Scale bar = 100 µm.

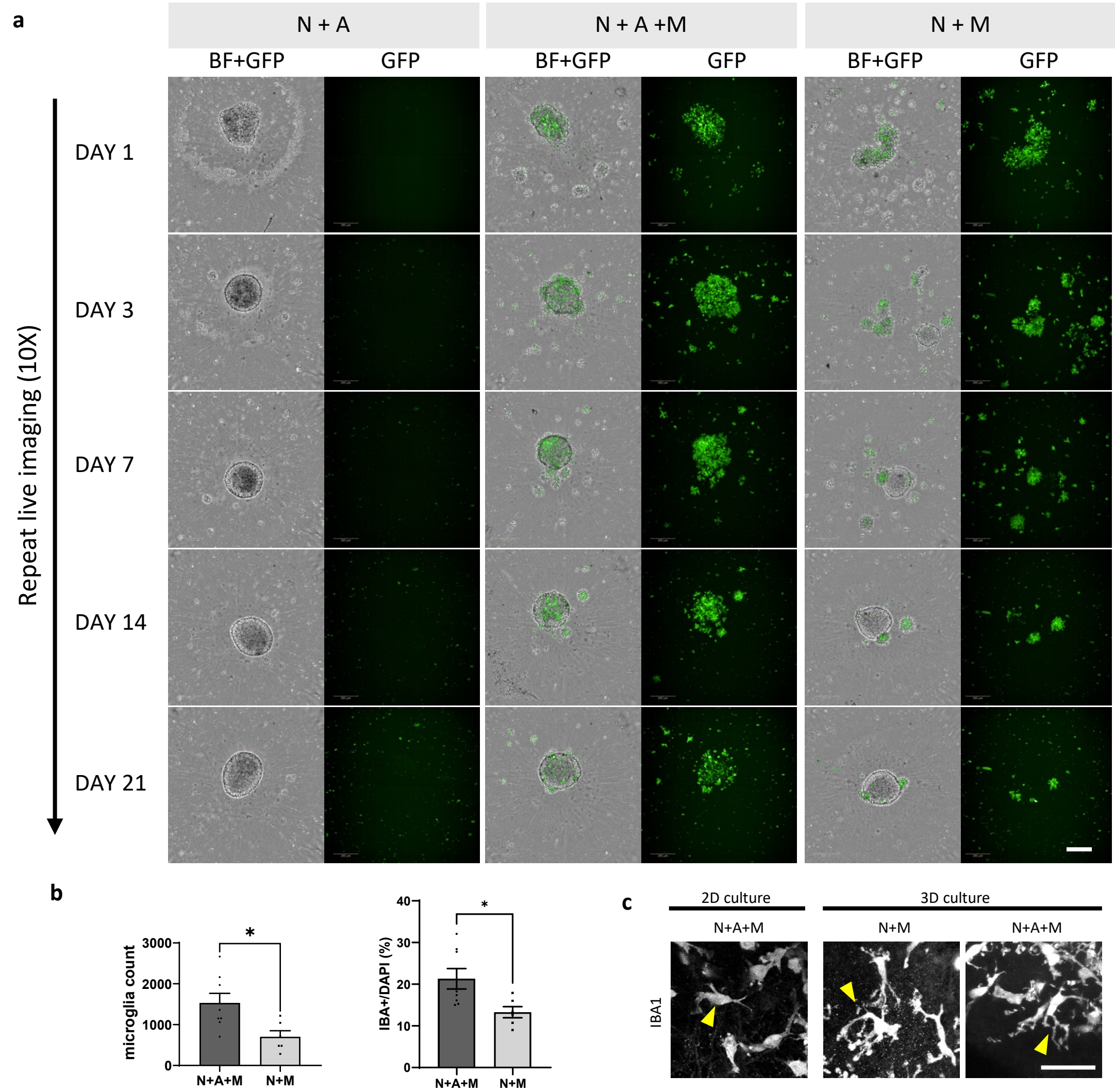

Supplementary Figure 3. The presence of astrocytes facilitates microglial integration into neural spheroids.
(a) Representative bright field (BF) or GFP-channel images of co-culture or tri-culture spheroids generated using mostly glutamatergic neurons (N, 10k/spheroid), together with astrocytes (A, 1k/spheroids) and/or GFP-tagged microglia (M, 2k/spheroid), captured via confocal live imaging using a 10X objective on DAY 1, 3, 7, 14, and 21. Each column shows images from the same spheroids. Each image was presented as maximum projection of a 150 µm z-stack. Scale bar = 200 µm.
(b) Left: Quantification of IBA1+ cells in N+A+M tri-culture and N+M co-culture spheroids. Right: Comparison of the percentage of microglia in total cell population between N+A+M and N+M spheroids. Data were collected from two independent experiments and four biological replicates on each experiment. Percentage was calculated by dividing the total number of IBA1+ cells by the total number of nuclei and presented as mean ± SEM. Error bars indicate the standard error of the mean. Asterisk symbol represents *$P < 0.05$.
(c) Confocal images of IBA1+ cells in 2D and 3D cell cultures. For 2D cultures, microglia (M) were seeded with glutamatergic neurons (Gl) and astrocytes (A) in coated 96w flat bottom plates as tri-cultures. For 3D cultures, microglia were seeded as glutamatergic neuron-microglia co-culture spheroids or tri-culture spheroids together with astrocytes. Representative images are presented as maximum projection of z-stacks covering the entire depth of the IBA1+ cells. Scale bar = 50 µm.

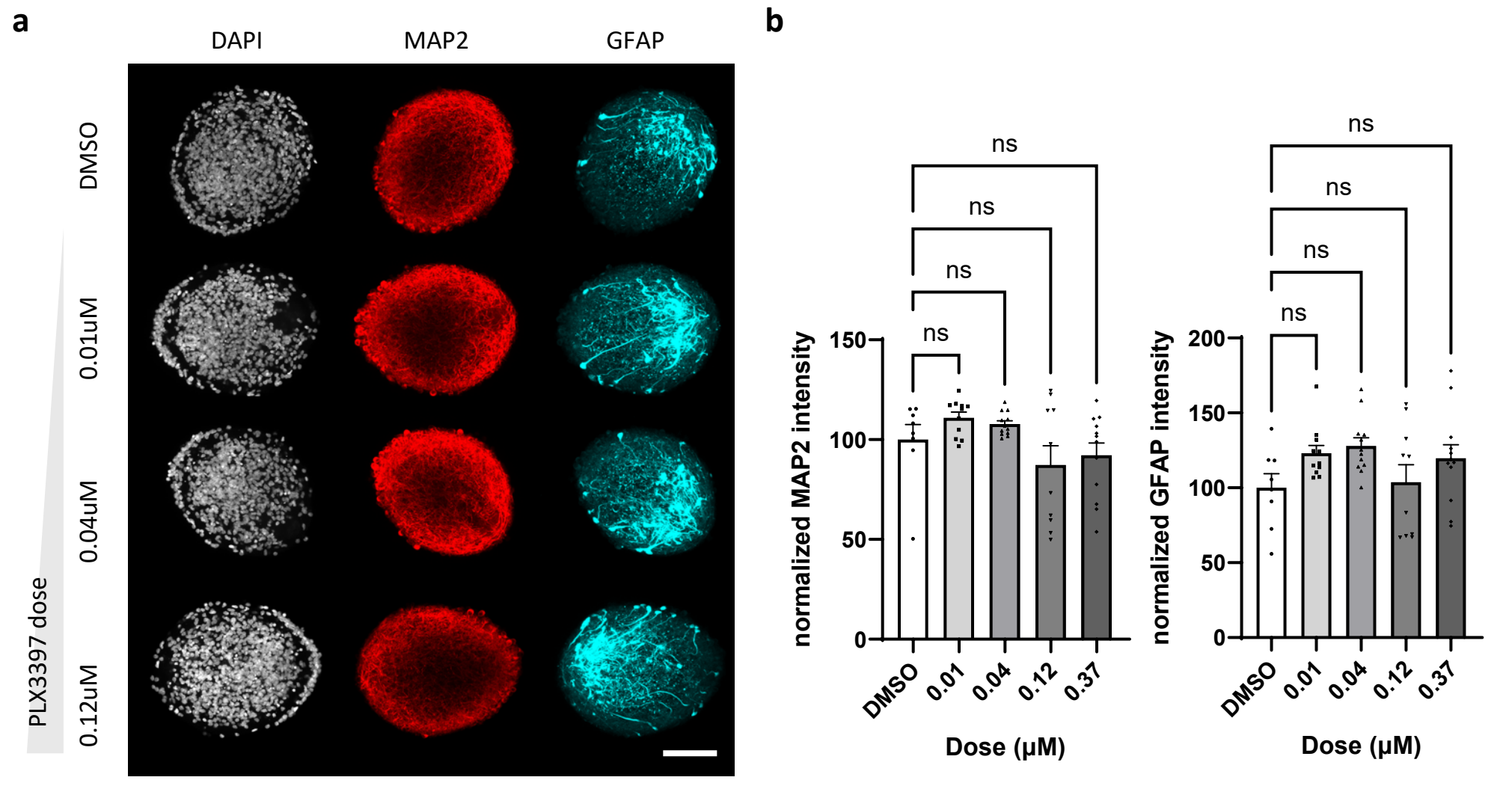

Supplementary Figure 4. PLX3397 treatment does not affect neuron or astrocyte viability in tri-culture spheroids.
(a) Confocal images of tri-culture spheroids after 96 hours of treatment with DMSO or PLX3397 (0.01, 0.04, or 0.12 µM). Spheroids were immunostained for MAP2 as neuronal marker and GFAP as astrocyte marker, followed by DAPI staining. Representative images are presented as maximum projections of 25 µm z-stacks. Scale bar = 100 µm.
(b) Quantification of MAP2 and GFAP signals in tri-culture spheroids treated with DMSO or PLX3397 (0.01, 0.04, or 0.12 µM) for 96 hours. Total integrated intensity over total volume was calculated and subsequently normalized to the DMSO group average. Statistical analysis was done using one-way ANOVA followed by Dunn's multiple comparisons test. Data were collected from three independent experiments with 4-8 biological replicates in each experiment and presented as mean ± SEM. Error bars indicate the standard error of the mean. ns = not significant.

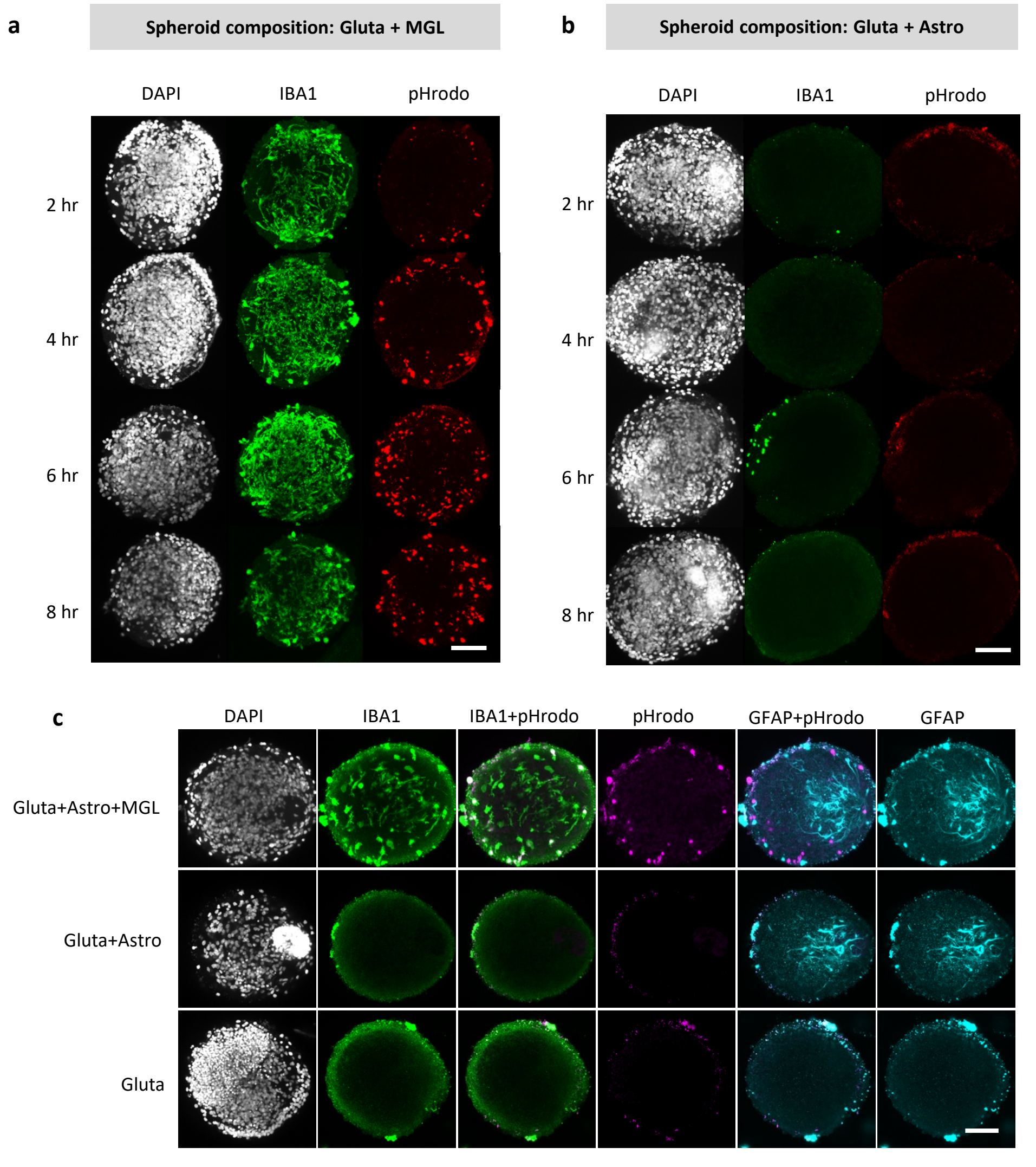

Supplementary Figure 5. Phagocytosis following pHrodo red *S. aureus* bioparticles exposure is mainly mediated by microglia.

(a) Confocal images of DAY 21 neuron-microglia co-culture spheroids fixed after a 2-, 4-, 6-, or 8-hour exposure to pHrodo red *S. aureus* bioparticles (100 µg/ml) and immunostained for IBA1 and counterstained for DAPI.

(b) Confocal images of DAY 21 neuron-astrocyte co-culture spheroids fixed after a 2-, 4-, 6-, or 8-hour exposure to pHrodo red *S. aureus* bioparticles (100 µg/ml) and immunostained for IBA1 and counterstained for DAPI.

(c) Confocal images of DAY 21 neuron-astrocyte-microglia tri-culture, neuron-astrocyte co-culture, or neuron monoculture spheroids fixed after an 8-hour exposure to pHrodo red *S. aureus* bioparticles (100 µg/ml) and immunostained for IBA1 or GFAP and counterstained for DAPI. Representative images are presented as maximum projections of 150 µm z-stacks. Scale bar = 100 µm.

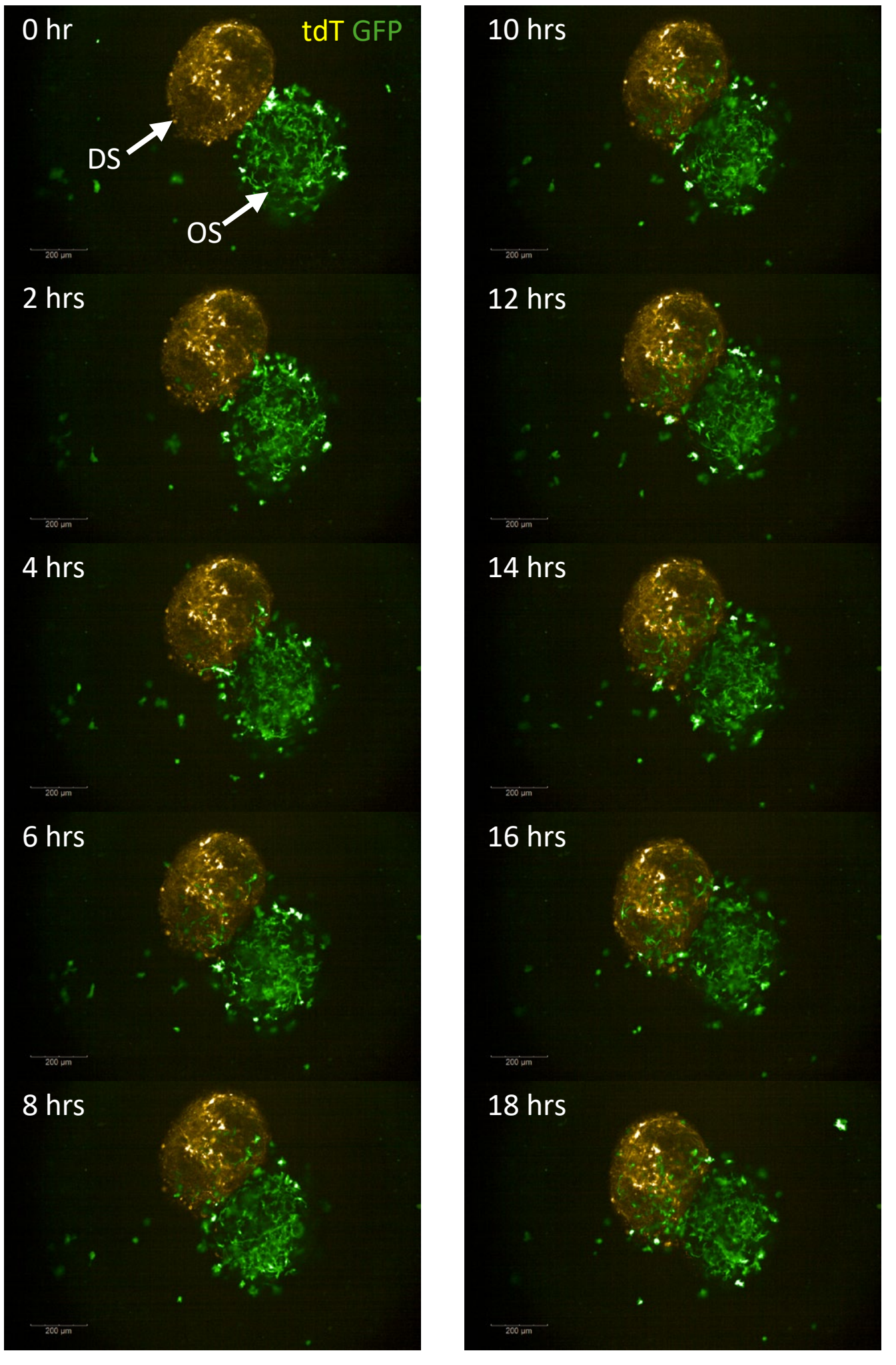

Supplementary Figure 6. **Migration of microglia in "origin spheroids" into "destination spheroids" in the assembloid-migration assay.**
Repeat live imaging of an assembloid made with one tdTomato (tdT)-expressing "destination spheroid (DS)" without microglia and one "origin spheroid (OS)" with GFP-tagged microglia. A single-plane image of the assembloid was taken using a Phenix spinning disk confocal with a 10X objective every 2 hours immediately following the generation of the assembloid. Scale bar = 200 µm.

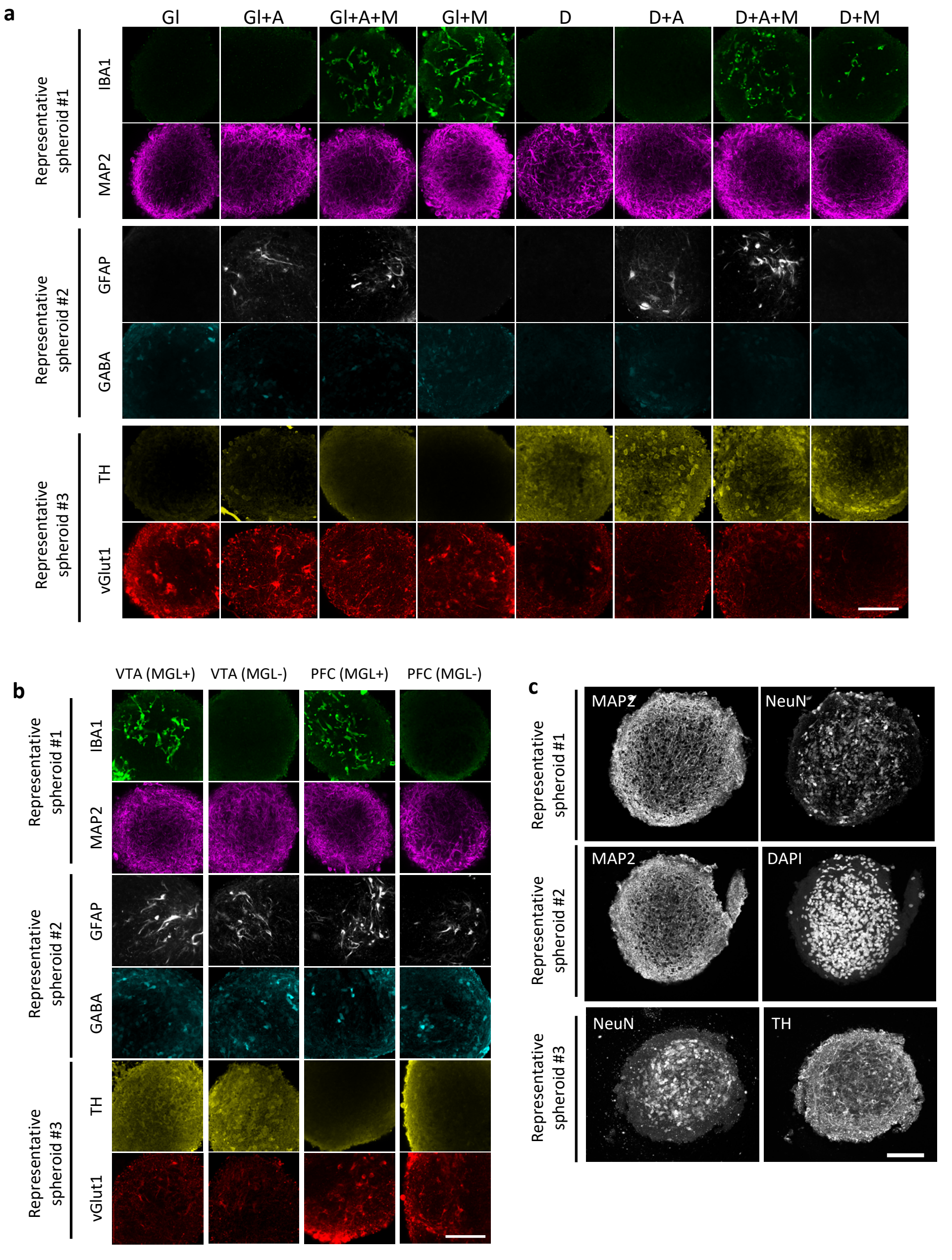

Supplementary Figure 7. Immunofluorescence-based characterization of neural spheroids seeded with different cell-type composition.

(a) Representative images (25X) of DAY 21 neural spheroids seeded with different cell-type compositions: mono-cultures of glutamatergic neurons (Gl) or dopaminergic neurons (D); co-cultures of neurons with astrocytes (A) or microglia (M); or tri-cultures of neurons, astrocytes, and microglia. Immunostaining of six cell-type markers were done in three separate groups of spheroids (top to bottom: spheroid group #1 stained for IBA1 and MAP2, spheroid group #2 stained for GFAP and GABA, spheroid group #3 stained for TH and vGlut1). Single-plane confocal images were taken using a 25X water objective and image acquisition settings were kept the same between samples. Scale bar = 100 µm.

(b) Representative images (25X) of DAY 21 brain-region-like spheroids seeded with or without 2k cells/spheroid of microglia: PFC-like spheroids (each seeded with 7k glutamatergic neurons + 3k GABAergic neurons + 1k astrocytes); VTA-like spheroids (each seeded with 6.5k dopaminergic neurons + 3k GABAergic neurons + 0.5k glutamatergic neurons + 1k astrocytes). Immunostaining of six cell-type markers were done in three separate groups of spheroids (top to bottom: spheroid group #1 stained for IBA1 and MAP2, spheroid group #2 stained for GFAP and GABA, spheroid group #3 stained for TH and vGlut1). Single-plane confocal images were taken using a 25X water objective and image acquisition settings were kept the same between samples. Scale bar = 100 µm.

(c) Representative images of DAY 21 VTA-like spheroids 30 µm cyrosections. Immunostaining of markers were done in three separate groups of sections (top to bottom: spheroid group #1 stained for MAP2 and NeuN, spheroid group #2 stained for MAP2 and DAPI, spheroid group #3 stained for NeuN and TH). Z-stack confocal images were taken using a 25X water objective to capture the entire section. Scale bar = 100 µm.

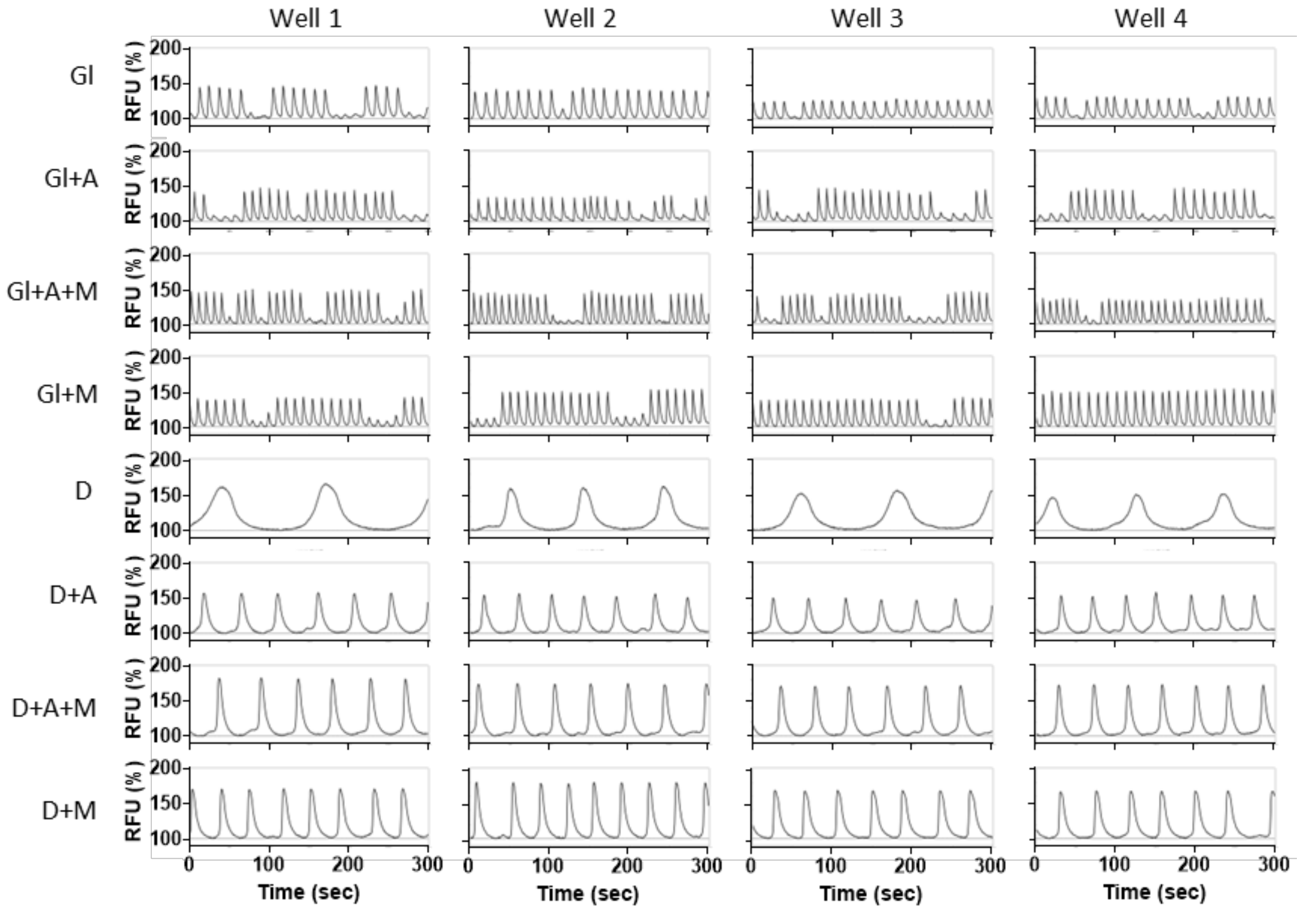

Supplementary Figure 8. Calcium oscillation profiles in single neuronal type spheroids cultured in tri-culture media. Spontaneous calcium oscillation traces of 4 spheroids generated in the same batch were shown for glutamatergic neurons only spheroids (Gl), dopaminergic neurons only spheroids (D), co-culture spheroids with astrocytes (Gl+A, D+A) or microglia (Gl+M, D+A), and tri-culture with astrocytes and microglia (Gl+A+M, D+A+M).

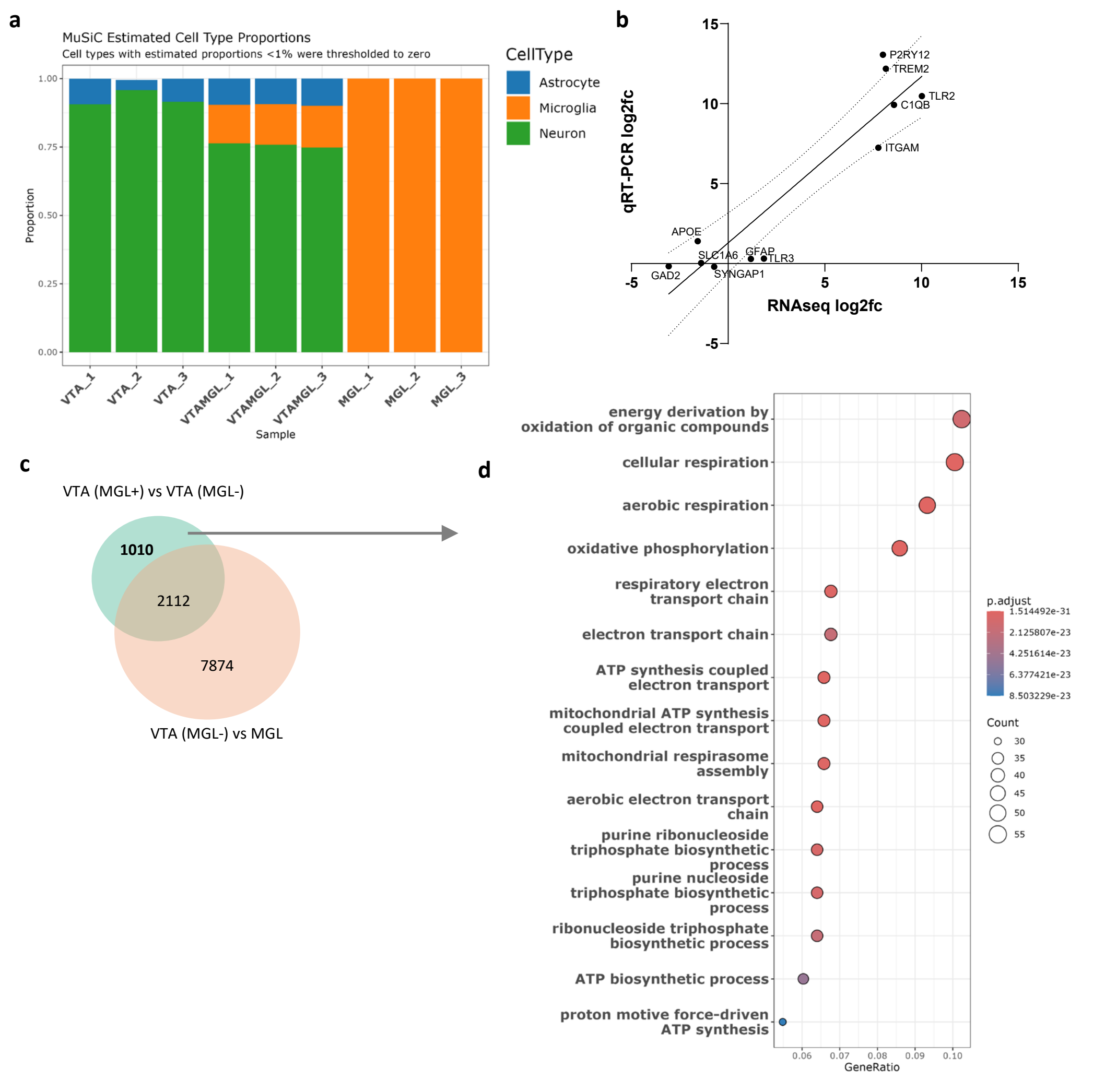

Supplementary Figure 9. Gene set enrichment analysis (GSEA) of a subset of DEGs between VTA-like spheroids with or without microglia and confirmation of cell populations.

(a) Confirmation of cell populations proportion using Multi-subject Single Cell (MuSiC) deconvolution.

(b) qRT-PCR vs. RNAseq log2fc DEGs of VTA (MGL-) vs. VTA(+MGL) neural spheroids. Pearson correlation co-effients computed assuming data are sampled from Gaussian distribution. Two-tailed p-value. Confidence interval 95%. r=0.9270, p<0.0001

(c) Venn diagrams showing significantly differentially expressed genes (DEGs) between VTA-like spheroids with (MGL+) or without (MGL-) microglia, using a threshold of log2 fold change >1 and p < 0.05 significance.

(d) Top 15 pathways identified using GSEA associated with genes that are differentially expressed in VTA-like spheroids due to microglia present, excluding genes that are also differentially expressed between VTA only and MGL only samples.

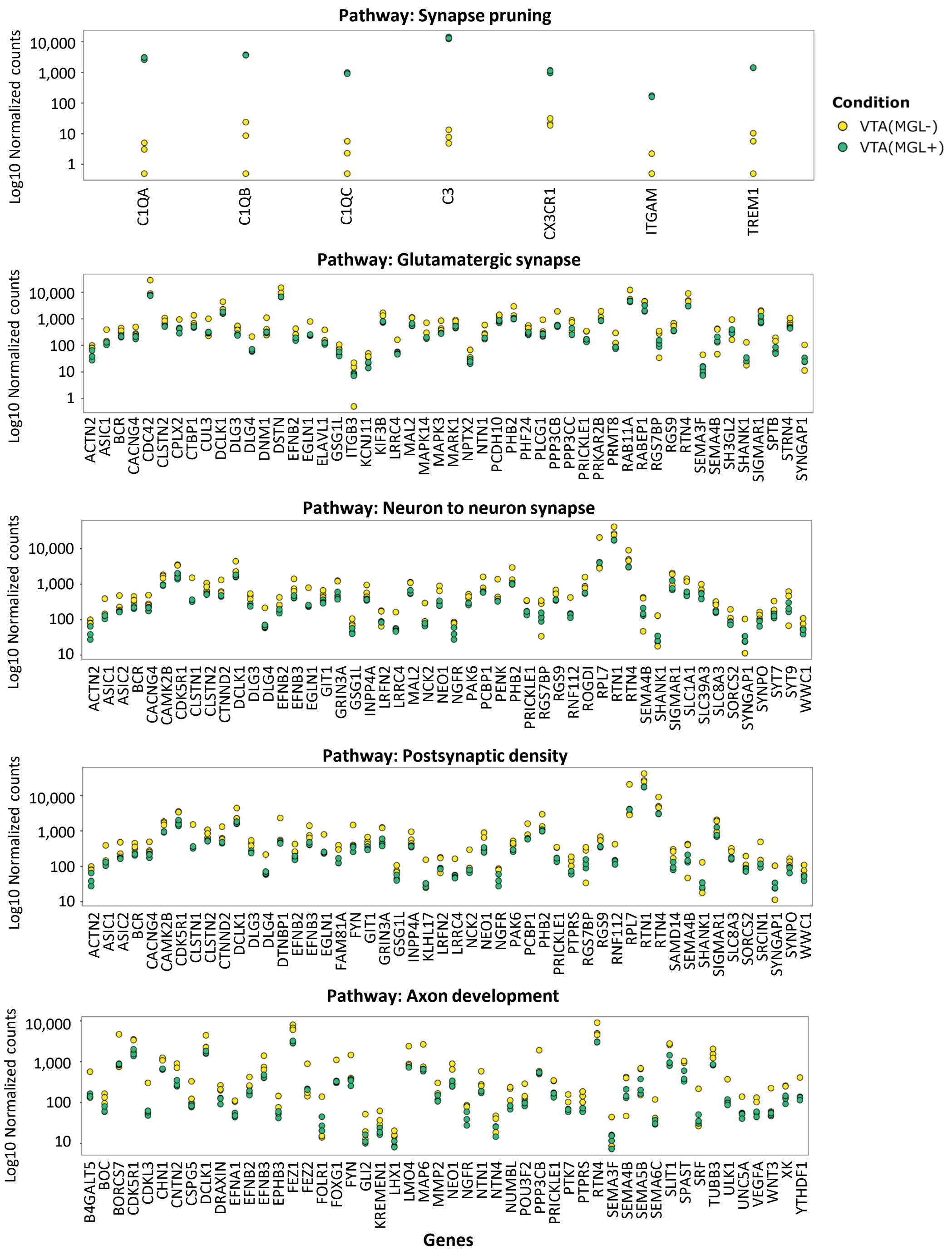

Supplementary Figure 10. **Gene panels enriched in GSEA of selected pathways between VTA-like spheroids and microglia only cultures.**

Top identified genes in selected GSEA pathways shown in Figure 4d.

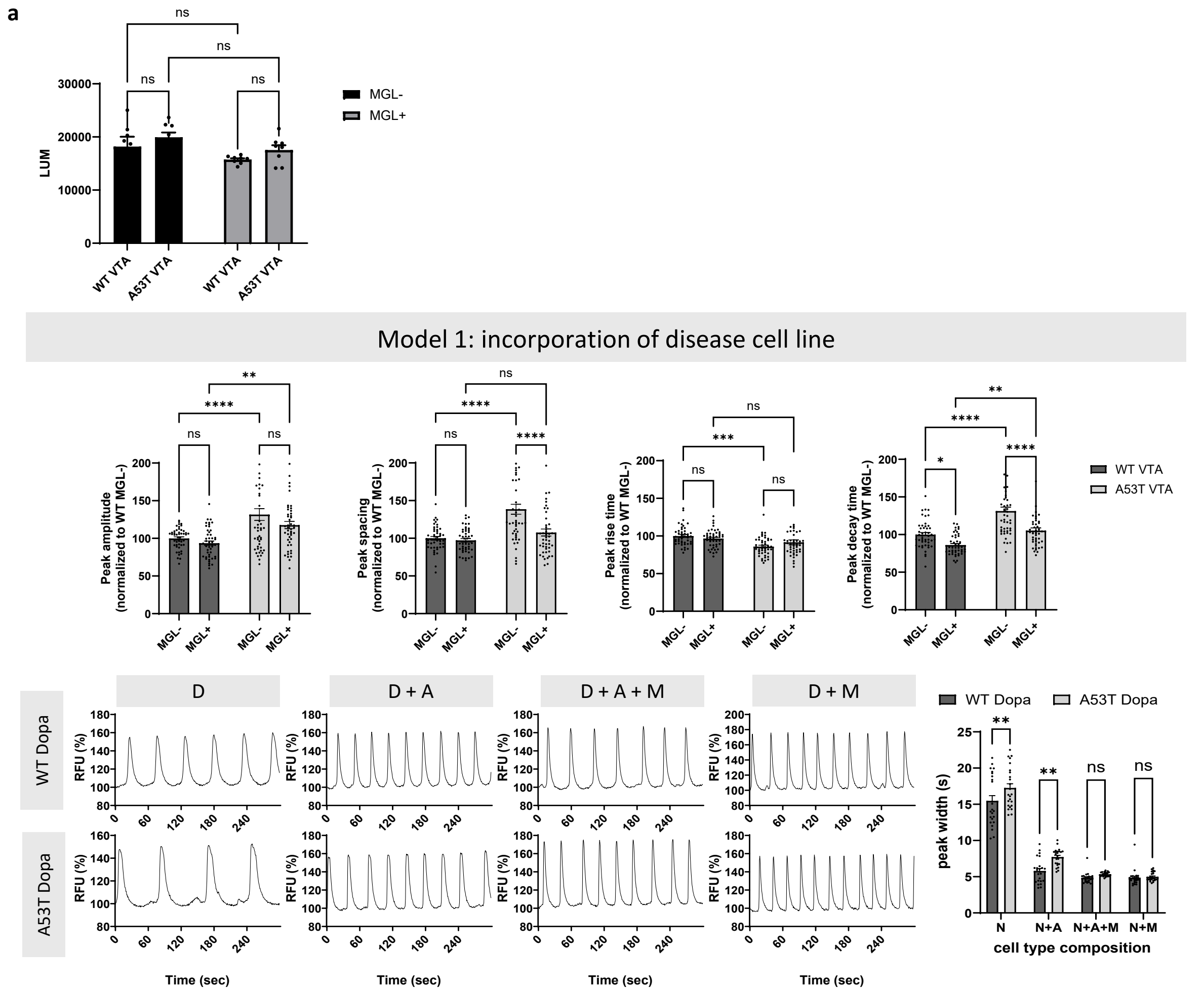

Supplementary Figure 11. Preliminary exploration of microglia-mediated disease phenotype attenuation in A53T VTA-like spheroids.

(**a**) Viability of DAY21 WT or A53T VTA-like spheroids were measure using the Cell Titer Glo-3D assay, where equal volumes of Cell Titer Glo-3D reagent was added to each spheroid-containing well, followed by vigorous mixed for 5 min and 25 min incubation at room temperature. Luminescence was read using a PHERAstar FSX microplate reader (BMG LabTech) to measure the amount of adenosine 5′-triphosphate (ATP) present, and the result suggested that the incorporation of A53T-dopaminergic neurons did not significantly changes the viability of the spheroids. Data were collected from one experiment with 8 biological replicates and presented as mean ± SEM.

(**b**) Data matching those used in Figure 6d to generate radar plots. Quantification of peak amplitude, peak spacing, peak rise time, and peak decay time using data collected from three independent experiments and 16 biological replicates in each experiment, and presented as mean ± SEM. Two-way ANOVA followed by Šídák's multiple comparisons test were used. Asterisk symbol represents *P < 0.05, **P < 0.01, ***P < 0.001, ****P < 0.0001, and ns = not significant.

(**c**) Spontaneous calcium oscillation traces of WT or A53T dopaminergic neurons spheroids seeded as neurons only (D), co-culture (D+A, D+M), or tri-culture (D+A+M) spheroids and quantification analysis of peak width. Error bars indicate the standard error of the mean.

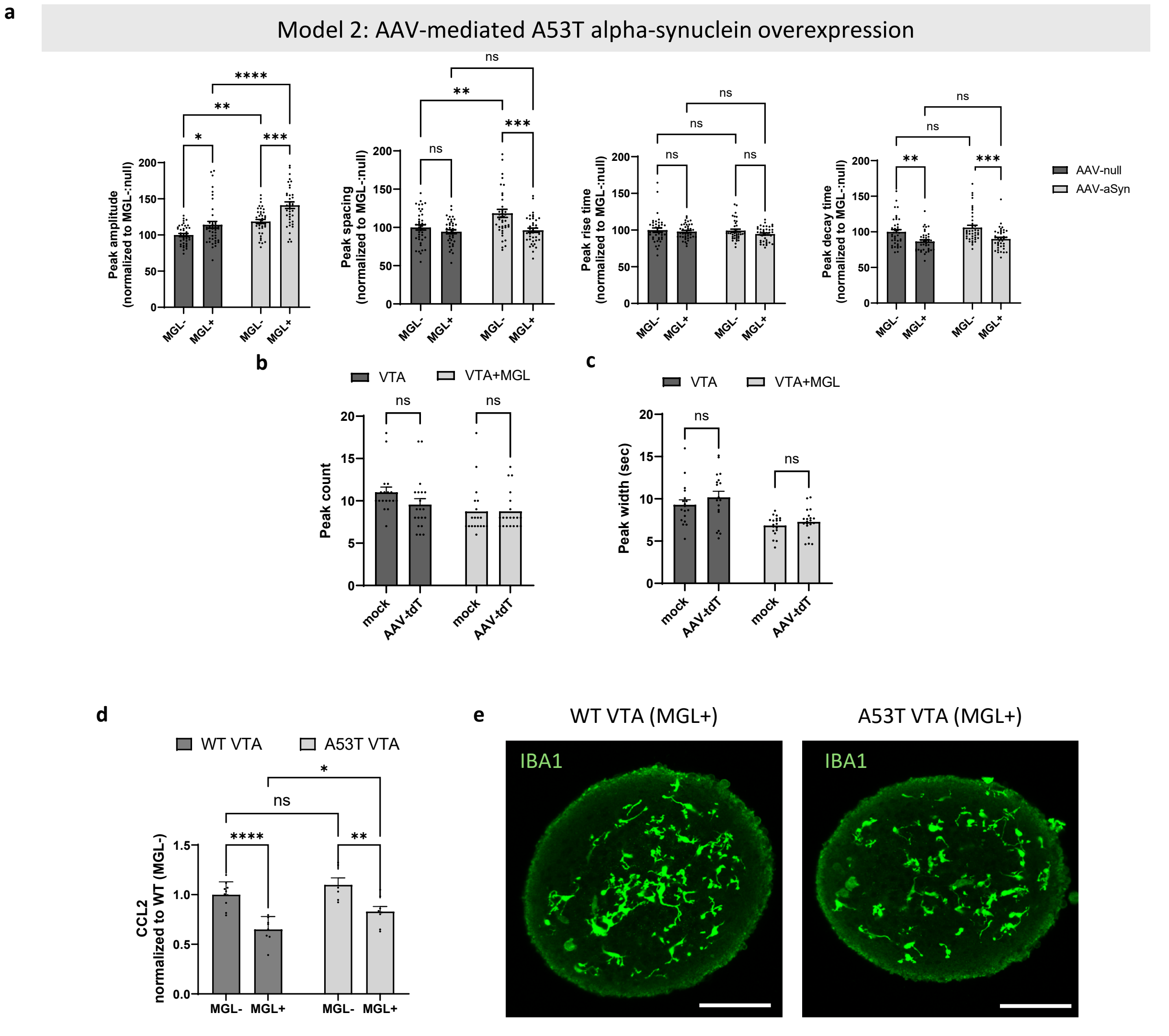

Supplementary Figure 12. Analysis of key peak parameters changes in AAV-A53T transduced VTA-like spheroids and preliminary exploration of microglia-mediated disease phenotype attenuation in A53T VTA-like spheroids.
(a) Data matching those used in Figure 5i to generate radar plots. Quantification of peak amplitude, peak spacing, peak rise time, and peak decay time using data collected from three independent experiments and 16 biological replicates in each experiment, and presented as mean ± SEM. Two-way ANOVA followed by Šídák's multiple comparisons test were used. Asterisk symbol represents *P < 0.05, **P < 0.01, ***P < 0.001, ****P < 0.0001, and ns = not significant.
(b-c) WT VTA with or without microglia were transduced with AAV2-CAG-tdTomato (MOI=5e5) as a control for AAV-mediated overexpression of A53T-alpha-synuclein on DAY 5 and their calcium oscillation was evaluated using Cal6-FLIPR assay on DAY 21. Data were collected from two independent experiments with 4-8 biological replicates in each experiment and presented as mean ± SEM. Two-way ANOVA followed by Šídák's multiple comparisons test was used. ns = not significant.
(d) Production of CCL2 detected in the supernatant of WT or A53T VTA-like spheroids with or without microglia (MGL) using Luminex multiplex assay. **(e)** Representative images showing maximum projections of a 10 µm sections z-stack in the middle of a WT VTA (MGL+) spheroid and a A53T VTA (MGL+) after 21 days in culture. Scale bar = 100 µm.
Error bars indicate the standard error of the mean.

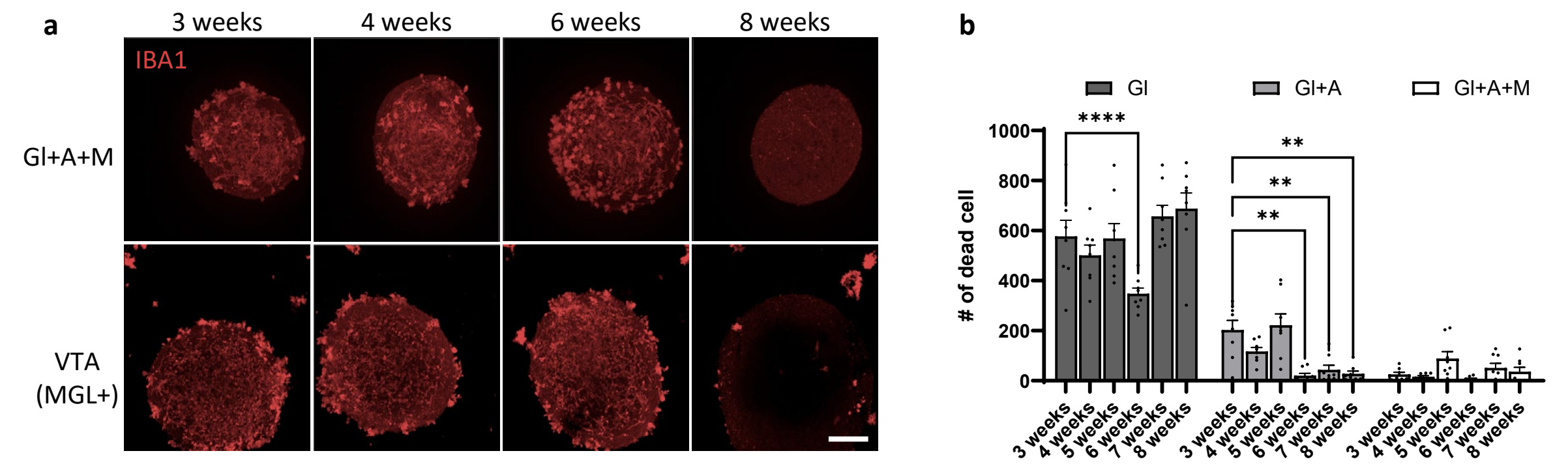

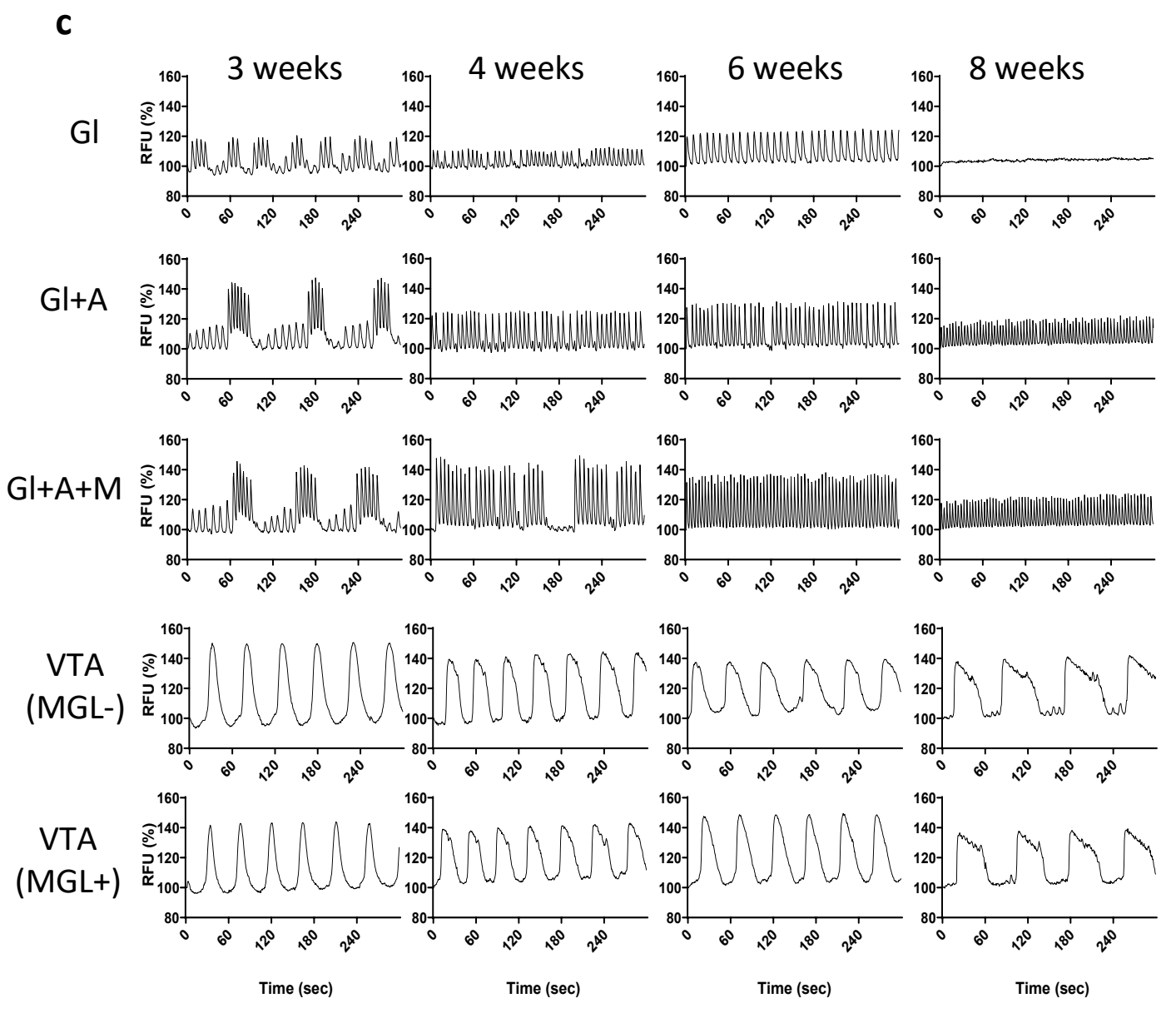

d

VTA(MGL-)				
	3 weeks	4 weeks	6 weeks	8 weeks
n=	24	24	24	24
PkRate	8.84%	19.58%	30.99%	80.27%
PkSp	8.62%	15.95%	29.53%	36.28%
PkWidth	11.33%	18.79%	38.68%	40.36%
PkA	31.92%	19.64%	22.11%	38.67%

VTA(MGL+)				
	3 weeks	4 weeks	6 weeks	8 weeks
n=	24	24	24	24
PkRate	8.03%	22.99%	25.50%	44.98%
PkSp	7.88%	18.40%	19.73%	28.27%
PkWidth	5.89%	16.80%	27.77%	31.28%
PkA	19.00%	24.29%	22.20%	24.80%

Supplementary Figure 13. Extended time course of monoculture, co-culture, or tri-culture spheroids.
(**a**) Representative images showing maximum projections of whole glutamatergic NeuroMIMICS or VTA-like NeuroMIMICS fixed after 3-, 4-, 6-, or 8-weeks in culture and stained for IBA1. Scale bar = 100 µm.
(**b**) Quantification of dead cells per spheroid over 3-,4-,6-, or 8-weeks culture. Error bars indicate the standard error of the mean.
(**c**) Representative calcium oscillation profiles in either monoculture neural spheroids, co-culture neural spheroids, or tri-culture NeuroMIMICS over 3-,4-,6-, or 8-weeks culture.
(**d**) Coefficient of Variation scores (%CV) for select peak parameters over time in VTA-like spheroids with or without microglia (MGL) after 3-, 4-, 6-, or 8-weeks in culture.

a

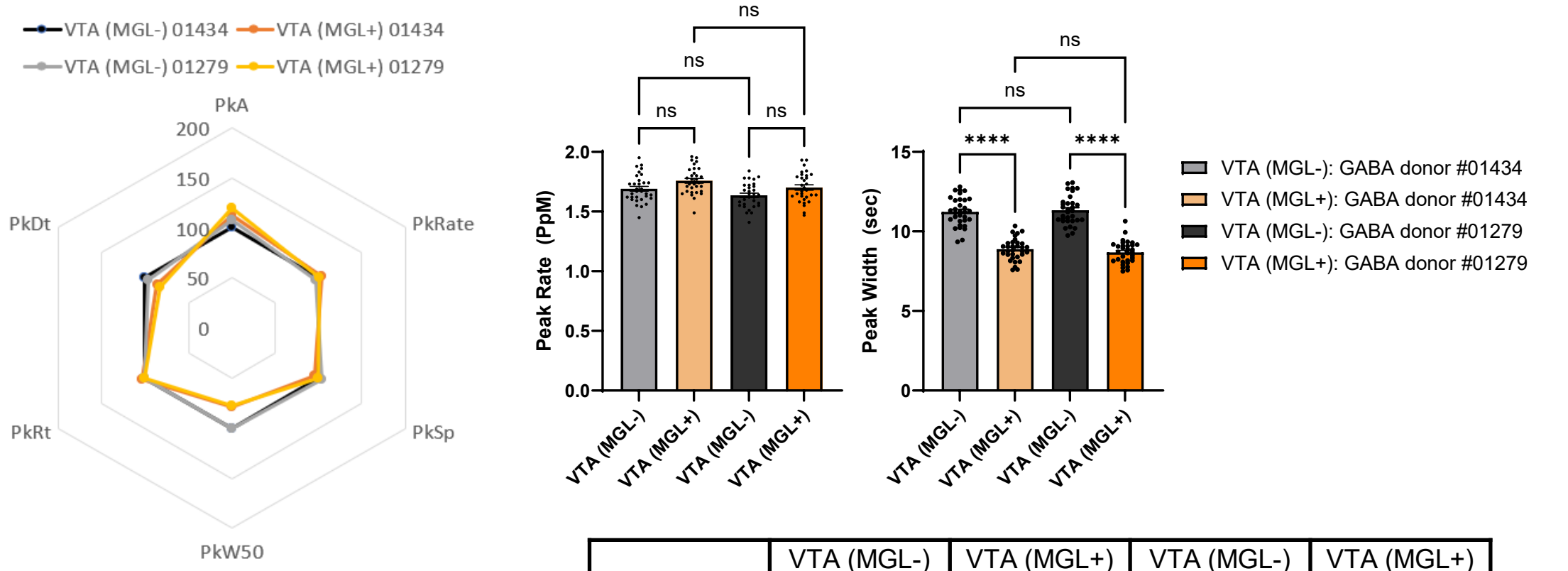

	VTA (MGL-) 01434	VTA (MGL+) 01434	VTA (MGL-) 01279	VTA (MGL+) 01279
Peak Rate	6.781%	6.318%	6.239%	7.143%
Peak Width	8.075%	7.801%	8.111%	8.164%

b

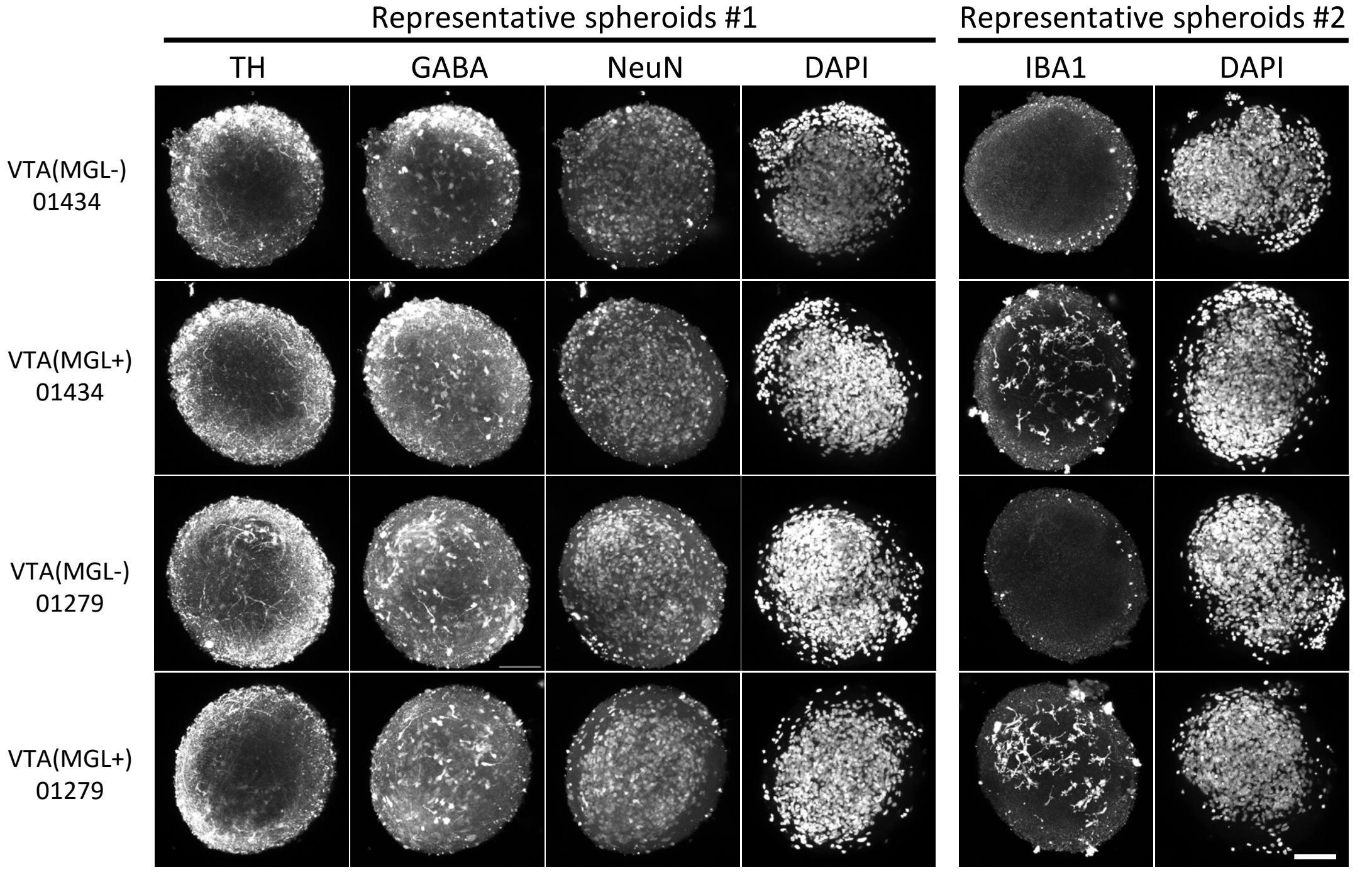

Supplementary Figure 14. **Incorporation of GABAergic neurons from two distinct healthy donors does not impact neural spheroid activity or composition.**
(**a**) Quantification of peak parameters and reproducibility from VTA-like spheroids with or without microglia (MGL) generated with GABAergic neurons from either donor 01434 or donor 01279. Error bars indicate the standard error of the mean.
(**b**) Representative images of DAY 21 VTA-like spheroids seeded with GABAergic neurons from donor 01434 or 01279. Immunostaining were done in two separate groups of spheroids: spheroid group #1 stained for TH, GABA, or NeuN, with DAPI for nuclei or spheroid group #2 stained for IBA1 and DAPI for nuclei. A z-stack of 100 µm confocal images were taken using a 25X water objective and image acquisition settings were kept the same between samples. Scale bar = 100 µm.

Supplementary Table 1. Average coefficient of variations (%CV) for neural spheroids.

	spheroid type	PkA	PkCt	PkRate	PkSp	PkW50	PkW90	RiseSlope	RiseTime	DecaySlope	DecayTime
**Figure 3**	D	18.31%	15.27%	14.72%	14.29%	15.91%	11.24%	32.73%	25.42%	20.44%	9.42%
	D+A	13.42%	14.09%	16.46%	12.58%	13.15%	8.56%	21.29%	15.72%	14.40%	5.75%
	D+A+M	16.32%	15.46%	16.43%	16.48%	7.73%	6.01%	23.46%	14.79%	15.34%	4.56%
	D+M	18.37%	12.84%	10.99%	10.76%	18.16%	10.38%	23.64%	12.58%	19.69%	7.42%
	Gl	13.13%	29.24%	15.90%	32.37%	18.10%	19.42%	20.24%	17.08%	16.57%	25.86%
	Gl+A	20.52%	27.82%	19.49%	24.55%	33.26%	21.84%	28.06%	17.46%	23.41%	11.10%
	Gl+A+M	30.19%	25.76%	13.54%	33.23%	14.61%	11.77%	46.50%	25.16%	30.22%	7.92%
	Gl+M	13.05%	13.70%	7.31%	12.95%	20.00%	18.63%	16.87%	10.37%	12.01%	4.94%
	VTA (MGL-)	11.71%	17.18%	16.70%	14.32%	17.46%	12.90%	12.68%	14.21%	17.28%	16.96%
	VTA (MGL+)	14.64%	18.33%	18.64%	16.86%	16.88%	12.72%	15.96%	12.04%	15.91%	15.27%
	PFC (MGL-)	10.14%	26.02%	16.60%	55.59%	20.18%	16.20%	13.80%	15.18%	14.14%	14.75%
	PFC (MGL+)	15.55%	38.65%	13.77%	33.27%	14.21%	13.04%	24.74%	25.68%	16.67%	10.33%
**Figure 6**	WT VTA (MGL-)	14.14%	17.86%	17.82%	16.17%	20.67%	14.79%	14.63%	12.66%	16.55%	18.30%
	WT VTA (MGL+)	20.56%	16.73%	16.86%	16.43%	15.04%	12.26%	19.27%	11.34%	17.27%	13.65%
	A53T VTA (MGL-)	17.80%	24.25%	24.41%	18.10%	20.92%	16.71%	16.89%	12.10%	17.15%	18.60%
	A53T VTA (MGL+)	19.58%	20.53%	21.07%	21.14%	24.20%	19.63%	17.51%	18.74%	17.89%	20.21%
	WT VTA (MGL-) : AAV-null	13.67%	22.51%	23.53%	22.34%	25.21%	19.61%	17.43%	19.17%	14.35%	19.99%
	WT VTA (MGL+) : AAV-null	11.75%	17.17%	15.34%	14.15%	13.97%	11.06%	12.61%	8.25%	13.13%	13.67%
	WT VTA (MGL-) : AAV-A53T	14.69%	17.29%	19.44%	18.53%	23.85%	17.91%	18.41%	12.64%	15.32%	13.57%
	WT VTA (MGL+) : AAV-A53T	15.22%	15.21%	14.23%	14.41%	15.77%	11.81%	18.13%	8.14%	13.42%	14.14%

Supplementary Table 2. Well-to-well viability of tri-culture spheroids.

	Batch 1	Batch 2	Batch 3
**Volumn (mm^3)**	0.017975	0.0242145	0.0181625
	0.017094	0.0251916	0.0174529
	0.019786	0.0260958	0.0178069
	0.017672	0.0234639	0.0164774
		0.0303547	0.0161329
			0.018853
**mean ± SD**	0.0181± 0.0012	0.0258±0.0027	0.0174±0.001
**%CV**	6.405	10.438	5.878

Supplementary Table 3. List of primary and secondary antibodies.

Antibody	Host	Manufacturers	Cat#	Dilution
anti-IBA1	rabbit	FUJIFILM Wako Pure Chemical Corporation	019-19841	1:500
anti-P2RY12	rabbit	Millipore Sigma	HPA014518	1:50
anti-MAP2	mouse	invitrogen	13-1500	1:1000
anti-GFAP	rat	Abcam	ab279291	1:1000
anti-GABA	rabbit	Millipore Sigma	A2052	1:500
anti-TH	chicken	Abcam	ab76442	1:500
anti-vGlut1	mouse	Abcam	ab242204	1:200
anti-CD68	mouse	ThermoFisher	MA5-13324	1:100
anti-alpha synuclein	mouse	BD Biosciences	610787	1:500
anti-alpha synuclein aggregate	rabbit	Abcam	ab209538	1:1000
Goat anti-Chicken IgY (H+L) Cross-Adsorbed Secondary Antibody, Alexa Fluor™ Plus 488	goat	Invitrogen	A32931	1:600
Goat anti-Rabbit IgG (H+L) Cross-Adsorbed Secondary Antibody, Alexa Fluor™ 568	goat	Invitrogen	A11011	1:600
Goat anti-Rabbit IgG (H+L) Cross-Adsorbed Secondary Antibody, Alexa Fluor™ 488	goat	Invitrogen	A11008	1:600
Goat anti-Rabbit IgG (H+L) Cross-Adsorbed Secondary Antibody, Alexa Fluor™ 647	goat	Invitrogen	A21244	1:600
Goat anti-Mouse IgG (H+L) Cross-Adsorbed Secondary Antibody, Alexa Fluor™ 647	goat	Invitrogen	A21235	1:600
Goat anti-Mouse IgG (H+L) Cross-Adsorbed Secondary Antibody, Alexa Fluor™ 568	goat	Invitrogen	A11004	1:600
Goat anti-Rat IgG (H+L) Cross-Adsorbed Secondary Antibody, Alexa Fluor™ 647	goat	Invitrogen	A21247	1:600

Supplementary Table 4. List of primers used for qRT-PCR.

NCBI Gene Symbol	Assay ID	RefSeq #	Exon Location	Dye
APOE	Hs.PT.56a.3799446	NM_000041(1)	Exon 3-4	FAM
C1QB	Hs.PT.56a.20374814	NM_000491(1)	Exon 1-2	HEX
GAD2	Hs.PT.58.372056	NM_000818(2)	Exon 4-6	HEX
GFAP	Hs.PT.58.1057167	NM_001131019(3)	Exon 1-3	HEX
ITGAM	Hs.PT.58.415044	NM_000632(2)	Exon 5-6	FAM
P2RY12	Hs.PT.58.25665095	NM_022788(1)	Exon 1-2b	TexRd
SLC1A6	Hs.PT.58.50476205	NR_073589(4)	Exon 5-8a	FAM
SYNGAP1	Hs.PT.58.4622325	NM_006772(1)	Exon 16-17	FAM
TLR2	Hs.PT.58.26767404	NM_003264(1)	Exon 1-2	HEX
TLR3	Hs.PT.58.25887499.g	NM_003265(1)	Exon 4	HEX
TREM2	Hs.PT.58.40294042	NM_001271821(2)	Exon 1a-2	FAM
GAPDH	Hs.PT.39a.22214836	NM_002046(1)	Exon 2-3	Cy5
