# Supplementary material for: Microglia integrated neural spheroids enable neuroinflammatory responses and correct network dysfunction induced by alpha-synuclein mutation
Source: Commun Biol. 2026 Jul 15;9:1482. doi: 10.1038/s42003-026-10696-w (PMC13740307; doi:10.1038/s42003-026-10696-w)
Supplement: Supplementary file 2 [file 42003_2026_10696_MOESM2_ESM.pdf]

Description of Additional Supplementary Files

File name: Supplementary Data
Description: The source data behind the graphs in the paper.
