# Supplementary material for: Microglia integrated neural spheroids enable neuroinflammatory responses and correct network dysfunction induced by alpha-synuclein mutation
Source: Commun Biol. 2026 Jul 15;9:1482. doi: 10.1038/s42003-026-10696-w (PMC13740307; doi:10.1038/s42003-026-10696-w)
Supplement: Supplementary file 3 [file 42003_2026_10696_MOESM4_ESM.pdf]

nature portfolio

Corresponding author(s): Marc Ferrer and Emily M. Lee

Last updated by author(s): Apr 24, 2026

Reporting Summary

Nature Portfolio wishes to improve the reproducibility of the work that we publish. This form provides structure for consistency and transparency in reporting. For further information on Nature Portfolio policies, see our Editorial Policies and the Editorial Policy Checklist.

Statistics

For all statistical analyses, confirm that the following items are present in the figure legend, table legend, main text, or Methods section.

n/a	Confirmed	
☐	☒	The exact sample size (*n*) for each experimental group/condition, given as a discrete number and unit of measurement
☐	☒	A statement on whether measurements were taken from distinct samples or whether the same sample was measured repeatedly
☐	☒	The statistical test(s) used AND whether they are one- or two-sided<br>*Only common tests should be described solely by name; describe more complex techniques in the Methods section.*
☐	☒	A description of all covariates tested
☐	☒	A description of any assumptions or corrections, such as tests of normality and adjustment for multiple comparisons
☐	☒	A full description of the statistical parameters including central tendency (e.g. means) or other basic estimates (e.g. regression coefficient) AND variation (e.g. standard deviation) or associated estimates of uncertainty (e.g. confidence intervals)
☐	☒	For null hypothesis testing, the test statistic (e.g. *F*, *t*, *r*) with confidence intervals, effect sizes, degrees of freedom and *P* value noted<br>*Give P values as exact values whenever suitable.*
☒	☐	For Bayesian analysis, information on the choice of priors and Markov chain Monte Carlo settings
☒	☐	For hierarchical and complex designs, identification of the appropriate level for tests and full reporting of outcomes
☐	☒	Estimates of effect sizes (e.g. Cohen's *d*, Pearson's *r*), indicating how they were calculated

Our web collection on statistics for biologists contains articles on many of the points above.

Software and code

Policy information about availability of computer code

Data collection	Screenworks 5.1 (Molecular Devices), Harmony 5.1 (Perkin Elmer)
Data analysis	ScreenWorks 5.1 via the PeakPro 2.0 module, Graphpad Prism 9.1, PImageJ, Columbus Image Data Storage and Analysis

For manuscripts utilizing custom algorithms or software that are central to the research but not yet described in published literature, software must be made available to editors and reviewers. We strongly encourage code deposition in a community repository (e.g. GitHub). See the Nature Portfolio guidelines for submitting code & software for further information.

Data

Policy information about availability of data

All manuscripts must include a data availability statement. This statement should provide the following information, where applicable:

- Accession codes, unique identifiers, or web links for publicly available datasets
- A description of any restrictions on data availability
- For clinical datasets or third party data, please ensure that the statement adheres to our policy

RNAseq datasets generated during and/or analyzed during the current study will be deposited to a public repository. All other data supporting the findings of this study are available within the paper and its Supplementary Information.

Research involving human participants, their data, or biological material

Policy information about studies with human participants or human data. See also policy information about sex, gender (identity/presentation), and sexual orientation and race, ethnicity and racism.

Reporting on sex and gender	n/a
Reporting on race, ethnicity, or other socially relevant groupings	n/a
Population characteristics	n/a
Recruitment	n/a
Ethics oversight	n/a

Note that full information on the approval of the study protocol must also be provided in the manuscript.

Field-specific reporting

Please select the one below that is the best fit for your research. If you are not sure, read the appropriate sections before making your selection.

☒ Life sciences ☐ Behavioural & social sciences ☐ Ecological, evolutionary & environmental sciences

For a reference copy of the document with all sections, see nature.com/documents/nr-reporting-summary-flat.pdf

Life sciences study design

All studies must disclose on these points even when the disclosure is negative.

Sample size	Data used in main figures were each collected from at least three independent trials except for bulk RNAseq (n=3 independent sample pools from one trial) and cytokine analysis (two independent trials in which supernatants from 3 independent spheroids were pooled as 1 sample, n = 2 samples were collected from each trial). Specifically, data from Figure 1F, 2C, 2E, 2H were each collected from three independent experiments with n=4-8 technical replicates per experiment; data from Figure 3 was collected from three independent experiments with n= 16 technical replicates per experiment; data from Figure 6 was each collected from four independent experiments with n=8-16 technical replicates per experiment.
Data exclusions	If spheroids were not in the detection window at time of recording the wells were counted as empty. Outliers were identified by using Grubbs' test, and outlier values are provided in supplementary excel file.
Replication	Data used in main figures were each collected from at least three independent trials except for bulk RNAseq (n=3 independent sample pools from one trial) and cytokine analysis (two independent trials in which supernatants from 3 independent spheroids were pooled as 1 sample, n = 2 samples were collected from each trial). Specifically, data from Figure 1F, 2C, 2E, 2H were each collected from three independent experiments with n=4-8 technical replicates per experiment; data from Figure 3 was collected from three independent experiments with n= 16 technical replicates per experiment; data from Figure 6 was each collected from four independent experiments with n=8-16 technical replicates per experiment.
Randomization	n/a see below
Blinding	For the work done in 384w plates with FLIPR, we acquire the entire plate at once and analysis is run on the entire plate.

Reporting for specific materials, systems and methods

We require information from authors about some types of materials, experimental systems and methods used in many studies. Here, indicate whether each material, system or method listed is relevant to your study. If you are not sure if a list item applies to your research, read the appropriate section before selecting a response.

Materials & experimental systems

n/a	Involved in the study
☐	☒ Antibodies
☐	☒ Eukaryotic cell lines
☒	☐ Palaeontology and archaeology
☒	☐ Animals and other organisms
☒	☐ Clinical data
☒	☐ Dual use research of concern
☒	☐ Plants

Methods

n/a	Involved in the study
☒	☐ ChIP-seq
☒	☐ Flow cytometry
☒	☐ MRI-based neuroimaging

Antibodies

Antibodies used

All antibodies used are in Supplementary Table 3

Validation

Describe the validation of each primary antibody for the species and application, noting any validation statements on the manufacturer's website, relevant citations, antibody profiles in online databases, or data provided in the manuscript.

Eukaryotic cell lines

Policy information about cell lines and Sex and Gender in Research

Cell line source(s)

Cryopreserved human iPSC-derived iCell Neurons, iCell Astrocytes, and iCell Microglia were purchased from FUJIFILM Cellular Dynamics International (FCDI). All cells used in this study were provided by the manufacturer as fully differentiated and highly pure population of cells that was quality checked for purity and cell profile via flow cytometry and RNAseq prior to shipping. Cell lines used in this study includes two donors across cell lines used, based on commercial availability: Donor ID# 01434, Caucasian female (age < 18) with a healthy phenotype and Donor ID# 01279, Caucasian male (aged 50-59) with a healthy phenotype. Wildtype cell lines used includes: iCell Astrocytes (Cat# R1092, Donor ID# 01434, , differentiated from fibroblast), iCell GlutaNeurons (Cat# C1033, Donor ID# 01279, differentiated from blood-derived iPSCs), iCell GABANeurons (Cat# C1012, Donor ID# 01434 , differentiated from fibroblast), iCell DopaNeurons (Cat# C1028, Donor ID# 01279, differentiated from blood-derived iPSCs), and iCell Microglia (Cat# C1110, Donor ID# 01279, differentiated from blood-derived iPSCs using a protocol previously developed by Blurton-Jones laboratory [12]). Two vials of GFP-labeled iCell Microglia (no-cat, Donor ID# 01279) were purchased from FCDI for the live imaging experiments used for initial assay optimization only, data acquired from GFP-microglia were not used in quantification or statistical analysis.
To investigate Parkinson's disease (PD) related phenotype, iCell DopaNeurons PD SNCA A53T HZ (Cat# C1113, Donor ID# 01279) which encodes heterozygous A53T allelic variant in the gene for SNCA and isogenic to iCell DopaNeurons was used.

Authentication

Manufacturer provided QC

Mycoplasma contamination

n/a

Commonly misidentified lines
(See ICLAC register)

n/a

Plants

Seed stocks

n/a

Novel plant genotypes

n/a

Authentication

n/a
